# OsmiR319-OsPCF5 modulate resistance to brown planthopper in rice through association with MYB proteins

**DOI:** 10.1186/s12915-024-01868-3

**Published:** 2024-03-22

**Authors:** Bo Sun, Yanjie Shen, Lin Zhu, Xiaofang Yang, Xue Liu, Dayong Li, Mulan Zhu, Xuexia Miao, Zhenying Shi

**Affiliations:** 1 Key Laboratory of Insect Developmental and Evolutionary Biology, CAS Center for Excellence in Molecular Plant Sciences, Institute of Plant Physiology and Ecology, Chinese Academy of Sciences, Shanghai, 200032 China; 2 University of Chinese Academy of Sciences, Beijing, 100049 China; 3 National Engineering Research Center for Vegetables, Beijing Vegetable Research Center, Beijing Academy of Agriculture and Forestry Science, Beijing, 100097 People’s Republic of China; 4 Shanghai Key Laboratory of Plant Functional Genomics and Resources, Shanghai Chenshan Botanical Garden, Shanghai, 201602 China

**Keywords:** OsmiR319, Brown planthopper, TCP, MYB, Resistance

## Abstract

**Background:**

The brown planthopper (BPH) is a kind of piercing-sucking insect specific to rice, with the damage tops the list of pathogens and insects in recent years. microRNAs (miRNAs) are pivotal regulators of plant–environment interactions, while the mechanism underlying their function against insects is largely unknown.

**Results:**

Here, we confirmed that OsmiR319, an ancient and conserved miRNA, negatively regulated resistance to BPHs, with overexpression of OsmiR319 susceptible to BPH, while suppression of OsmiR319 resistant to BPH in comparison with wild type. Meanwhile, we identified several targets of OsmiR319 that may mediate BPH resistance. Among them, *OsPCF5* was the most obviously induced by BPH feeding, and over expression of *OsPCF5* was resistance to BPH. In addition, various biochemical assays verified that OsPCF5 interacted with several MYB proteins, such as OsMYB22, OsMYB30, and OsMYB30C.Genetically, we revealed that both OsMYB22 and OsMYB30C positively regulated BPH resistance. Genetic interaction analyses confirmed that *OsMYB22* and *OsMYB30C* both function in the same genetic pathway with OsmiR319b to mediate BPH resistance.

**Conclusions:**

Altogether, we revealed that OsPCF5 regulates BPH resistance via association with several MYB proteins downstream of OsmiR319, these MYB proteins might function as regulators of BPH resistance through regulating the phenylpropane synthesis.

**Supplementary Information:**

The online version contains supplementary material available at 10.1186/s12915-024-01868-3.

## Background

The brown planthopper (BPH) is a rice-specific piercing-sucking insect pest that causes catastrophic yield and economic losses each year in rice-planting areas [1]. While sucking juices from vascular tissue, BPHs not only directly dehydrate plants, but also transmit several viruses, such as grassy stunt virus and rugged stunt virus, and thus indirectly damage rice. The traditional approach to control BPHs is still heavily dependent on chemical pesticides, which are harmful to predators of this insect and cause residual environmental pollution. Promoting host defense by breeding resistant cultivars is therefore a more cost-effective and environmentally friendly method of integrated pest management. Among many quantitative trait locus–encoded BPH resistance genes identified in wild and cultivated rice, 17 have been successfully cloned through positional cloning [2–12]. However, the BPHs can quickly switch among different biotypes to circumvent existing endogenous resistance genes [11]. As a typical example, the resistance conferred by the *BPH1* gene in rice variety IR26 was overcome by the BPHs in only 3 years. Additional resistance genes and new integrated strategies are therefore needed. Therefore, several BPH resistance genes with broad-spectrum resistance have been cloned in recent years, with different mechanisms, such as the clustering of three lectin receptor kinase genes by *BPH3* [5], the allelic diversity of *BPH9/1/7/10/21* [9], and the fortification of sclerenchyma tissue and cell walls by *BPH30* [11].

MicroRNAs (miRNAs) are important regulators useful for crop improvement and protection against different adverse environmental factors [13]. In plants with BPH resistance mediated by *BPH15*, numerous miRNAs are differentially expressed compared with those in susceptible ones, and BPH infestation induces even more miRNAs to be differentially expressed between resistant and susceptible plants [14]. Similarly, many miRNA-mRNA pairs are involved the BPH6-mediated BPH resistance [15]. In general, BPH infestation induces or suppresses many miRNAs, including OsmiR396, which regulates rice resistance to BPHs by direct transcriptional regulation on *OsF3H* gene and accordingly modulating the flavonoid biosynthesis pathway [16]. In addition, OsmiR156 positively regulates rice resistance to BPHs through the jasmonate acid (JA) biosynthetic pathway [17]. OsmiR159 negatively regulate mediates BPH resistance through repressing G-protein γ subunit *GS3* by the GAMYBL2 target [18]. These preliminary studies indicate the active participation of rice miRNAs in plant defense against BPHs via various signaling pathways.

miR319 is an ancient, conserved miRNA regulating leaf development and immunity in plants. Three miR319 genes are present in *Arabidopsis *(http://structuralbiology.cau.edu.cn/PNRD). miR319, first identified in the *Arabidopsis*-dominant mutant *jaw-D* as a regulator of leaf development, primarily functions in marginal regions, and its disruption leads to uneven leaf development and leaf curvature [19, 20]. Meanwhile, miR319 regulates cell proliferation and cell cycle in cooperation with miR164 and miR396 [21–23]. Moreover, miR319 is involved in responses to various types of abiotic stress, such as cold [20], drought [24], and salinity [25] in different plant species. In *Arabidopsis*, miR319-controlled TEOSINTEBRANCHED1/CYCLOIDEA/PROLIFERATING CELL FACTOR (TCP) negatively regulates leaf growth and positively functions in leaf senescence by regulating JA biosynthesis [26]. Two members of OsmiR319 are found in rice (http://structuralbiology.cau.edu.cn/PNRD). Overexpression of OsmiR319 results in broadened leaf blades [20]. And OsmiR319 together with two of its target genes, OsTCP21 and OsGAmyb, regulate rice tiller bud development and thus influence grain yield [27]. In immunity, OsmiR319 coupled with its target gene, *OsTCP21*, can suppress JA biosynthesis and signaling to facilitate the infection of rice ragged stunt virus as well as the fungal pathogen *Magnaporthe oryzae* [28, 29], thus indicating the active participation of miR319 in plant immunity. In *Populus tomentosa*, miR319 together with its target, *TCP19*, coordinately regulate trichome initiation and mediate resistance to insects in association with gibberellin (GA) signaling [30]. Given these results, the possibility that OsmiR319 mediates resistance to BPHs is an intriguing idea.

TCP proteins, which belong to the bHLH transcription factor family, are key determinants of plant conformation. TCP proteins usually associate with V-myb myeloblastosis viral oncogene homolog (MYB) proteins, one of the largest plant transcription factor families [31, 32]. *MYB* genes function extensively in plant development and metabolic regulation [33–36]. In higher plants, specific R2R3-MYB proteins developmentally and environmentally regulate secondary metabolism, especially flavonoid biosynthesis, via the formation of MYB/bHLH/WD40 (MBW) complexes*.* The functions of more than 100 MYB proteins in phenylpropanoid metabolism have been studied in various plant species; however, few of them are from rice [34]. Basically, the MBW complex is conserved in rice to regulate flavonoid biosynthesis and hull pigmentation [37]. Specifically, OsMYBS1 could negatively regulate the negative immune regulator, *Bsr-d1*, by direct binding to its promoter, thus inhibit the degradation of H_2_O_2_ and promote resistance to rice blast disease [38]. *OsMYB30* positively modulates BPH resistance by regulating *phenylalanine ammonia-lyase6* (*OsPAL6*) and *OsPAL8*, which participate in the phenylpropanoid biosynthesis pathway and positively regulate BPH resistance by influencing lignin and salicylic acid (SA) contents [39]. There is another *OsMYB30* (LOC_Os02g41510) (https://www.ricedata.cn/gene/), which we have tentatively renamed as *OsMYB30C* to distinguish from *OsMYB30. OsMYB30* regulates cold tolerance through interaction with jasmonate ZIM-domain protein 9 (OsJAZ9) and fine-tuning of maltose content; meanwhile, *OsMYB30C* modulates blast resistance through regulation of 4-*coumarate:coenzyme A ligase3* (*Os4CL3*) and *Os4CL5*, resulting in the accumulation of lignin subunits G and S and thickening of sclerenchyma cells in the leaf epidermis [40, 41]. These studies have revealed the tight functional association of MYB proteins with secondary metabolism in rice. An interesting question is whether this association can mediate rice resistance to BPHs as well.

In this study, we found that *OsMIR319a* and *OsMIR319b* genes in rice were both responsive to BPH infestation. Overexpression of *OsMIR319a* and *OsMIR319b* resulted in susceptibility to BPHs, whereas OsmiR319 downregulation through overexpression of the target mimic miR319 (MIM319) conferred resistance to BPH. Among the putative target genes of OsmiR319, *OsPCF5* showed the most obvious response to BPH infestation. Moreover, *OsPCF5*-overexpressing plants were resistant to BPHs, suggesting the involvement of OsmiR319 and its target in BPH resistance. Biochemistry assays revealed that OsmiR319 may regulate BPH resistance through interaction with several MYB proteins, including OsMYB30, OsMYB30C, and OsMYB22. In addition to OsMYB30, which has been previously reported to regulate BPH resistance [39], we determined that OsMYB30C and OsMYB22 both positively regulate BPH resistance. Furthermore, both MYB22OE and MYB30COE were able to compliment the BPH-susceptible characteristics of miR319bOE plants. We have therefore revealed that the OsmiR319/OsPCF5 module regulates BPH resistance through an association with several MYB proteins, perhaps thereby guiding the rearrangement of secondary metabolism.

## Results

### Induced expression of OsmiR319a and OsmiR319b due to BPH infestation

**Fig. 1:**
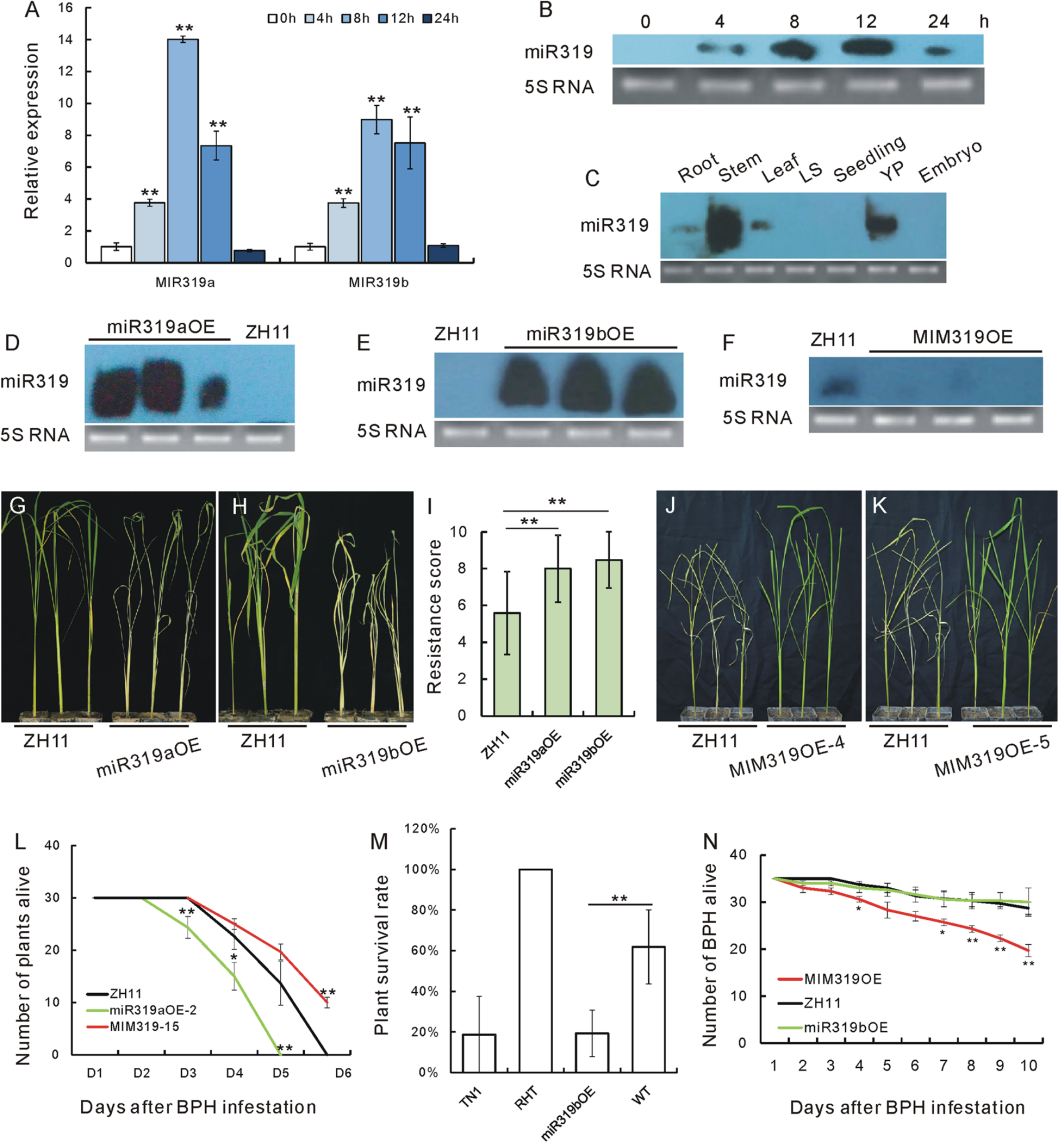
OsmiR319 expression and function in response to BPHs. **A** Expressions of *OsMIR319a* and *OsMIR319b* genes upon BPH infestation as revealed by qRT-PCR of pri-OsmiR319a and pri-OsmiR319b, respectively (*n* = 3). The expression level at 0 h was set as 1.0, and asterisks indicate significant differences comparing with 0 h as determined by Student’s *t* test (**, *P* < 0.01). Individual data values are provided in table S2. **B** Expression of OsmiR319 upon BPH infestation as revealed by miRNA Northern blotting. 5S RNA was used as a loading control. **C** Expression profile of OsmiR319 in different tissues of rice plants as revealed by miRNA Northern blotting. 5S RNA was used as a loading control. LS, leaf sheath, YP, 3-cm young panicles. Embryos were collected 10 days after fertilization. **D**–**F** miRNA Northern blot revealing the expression of OsmiR319 in miR319aOE, miR319bOE, and MIM319OE plants relative to ZH11 plants respectively. 5S RNA was used as a loading control. **G** Individual tests of miR319aOE and ZH11 plants to BPH infestation. **H** Individual tests of miR319bOE and ZH11 plants to BPHs. **I** Resistance scores of the WT, miR319aOE, and miR319bOE plants in the bulked seedling test (*n* = 10). Asterisks indicate significant differences compared with ZH11 plants as determined by Student’s *t* test (**, *P* < 0.01). **J**,**K** Individual tests of MIM319OE-4, MIM319OE-5, and ZH11 plants to BPH infestation. **L** Number of plants alive during a process of BPH feeding for 6 days (*n* = 3). Asterisks indicate significant differences compared with that of ZH11 plants as determined by Student’s *t* test (**, *P* < 0.01). Individual data values are provided in table S4. **M** Survival rates of different plant varieties and lines after BPH infestation (*n* = 3). Asterisks indicate significant differences as determined by Student’s *t* test (**, *P* < 0.01). The varieties RHT and TN1 were respectively used as resistant and susceptible controls. Individual data values are provided in table S5. **N** Number of BPHs alive on miR319bOE, MIM319OE, and ZH11 plants during 10 days of infestation (*n* = 3). Asterisks indicate significant differences compared with ZH11 plants as determined by Student’s *t* test (**, *P* < 0.01; *, *P* < 0.05). Individual data values are provided in table S6

Two genes encode OsmiR319 in rice: *OsMIR319a* and *OsMIR319b* (http://structuralbiology.cau.edu.cn/PNRD). We have previously shown that OsmiR319 overexpression enhances tolerance to cold stress [20]. Considering the functional diversity of miRNAs, OsmiR319 might also be involved in response to biological stresses. To test whether OsmiR319 participates in BPH resistance, we first measured the expression of OsmiR319 upon BPH infestation at different time points. According to the qRT-PCR results, pri-OsmiR319a and pri-OsmiR319b were both induced by BPH infestation, with the highest induction observed 8 h after infestation (Fig. 1A, Additional file 1A). A miRNA Northern blot assay further confirmed that mature OsmiR319 (both OsmiR319a and OsmiR319b, whose mature sequences are identical) was obviously accumulated upon BPH infestation, with the highest induction occurring around 8 and 12 h after infestation (Fig. 1B). This inductive expression profile strongly suggests that OsmiR319 is responsive to BPH feeding in rice.

To further investigate the possible involvement of OsmiR319 in BPH response, we profiled the expression of OsmiR319 in different rice plant tissues by Northern blotting. It revealed that OsmiR319 expressed highly in stems and young panicles (Fig. 1C).

### Negative regulation of rice BPH resistance by OsmiR319

To investigate the function of OsmiR319 in BPH resistance, we constructed *OsMIR319a*- and *OsMIR319b*-overexpressing transgenic lines (miR319aOE and miR319bOE, respectively) and the target mimic miR319 (MIM319)-overexpressing (MIM319OE) transgenic plants driven by the 35S promoter in the ZH11 genetic background. As revealed by miRNA Northern blotting, OsmiR319 was strongly upregulated in both miR319aOE and miR319bOE transgenic plants (Fig. 1D, E), but obviously downregulated in MIM319OE plants (Fig. 1F). Consistent with the phenotypes of corresponding plants in the Kasalath genetic background [20], OsmiR319 overexpression plants had widened leaves, while the MIM319OE plants had narrowed leaves (Additional file 2A). Accordingly, the leaf length/width ratio of miR319aOE plants was lower, while that of MIM319OE plants was higher, than that of the wild type (WT) (Additional files 1B and  2B), thus further verifying the positive role of OsmiR319 in regulating the characteristics of rice leaf blades.

We next used the miR319aOE, miR319bOE, and MIM319OE plants to investigate the genetic functions of OsmiR319a and OsmiR319b in response to BPHs. When individual plants were tested for their response to BPH infestation, miR319aOE and miR319bOE plants died earlier than WT ones, which indicates the susceptibility of these transgenic plants to BPHs (Fig. 1G, H). Consistently, in bulked seedling test, the resistance scores of miR319aOE and miR319bOE were both higher than that of WT (Fig. 1I), also indicating a susceptible character of miR319aOE and miR319bOE plants. In contrast, MIM319OE plants succumbed later than the WT, thus indicating their resistance characteristic to BPHs (Fig. 1J, K). We subsequently carried out small population assays, which confirmed that miR319aOE and miR319bOE plants died earlier than the WT (Additional file 3A, B), while MIM319OE plants died later (Additional file 3C, D) than the WT. By comparing the pace of plants dying upon BPH infestation, we found that more miR319aOE plants died than the WT in a much quicker way, while fewer MIM319OE plants died than the WT during BPH infestation in a much slower way (Fig. 1L, Additional file 1C), which further demonstrated the negative regulation of BPH resistance by OsmiR319. We also compared the response of miR319bOE plants to BPHs with that of TN1, a highly susceptible variety, which revealed that the susceptibility level of miR319OE was comparable to that of TN1 (Fig. 1M, Additional file 1D).

Plants have adopted three resistance strategies against insects: antixenosis to affect insect settling, colonization, and oviposition; antibiosis to reduce insect survival rates or feeding activity; and tolerance to withstand insect damage [42]. To determine the mechanism of miR319bOE and MIM319OE plant response to BPHs, we counted the number of BPHs on miR319bOE, MIM319OE, and WT plants. The number of BPHs on MIM319OE plants decreased quickly as feeding progressed, much faster than on WT and miR319bOE plants (Fig. 1N, Additional file 1E). This result indicates that antibiosis accounts for the mechanism of OsmiR319-mediated BPH resistance.

At the same time, we overexpressed the *OsMIR319a* gene in the TP309 genetic background, and the *OsMIR319b* gene in TP 309 and Kasalath genetic backgrounds to yield transgenic plants miR319aOET, miR319bOET, and miR319bOEK, respectively. Under BPH infestation, miR319aOET, miR319bOET, and miR319bOEK plants died earlier than their respective WTs (Additional file 4A-C). These results using plants from various genetic backgrounds further confirmed that OsmiR319 negatively regulates plant response to BPH infestation.

### Expression of target genes of OsmiR319 and functional analysis of OsPCF5

**Fig. 2:**
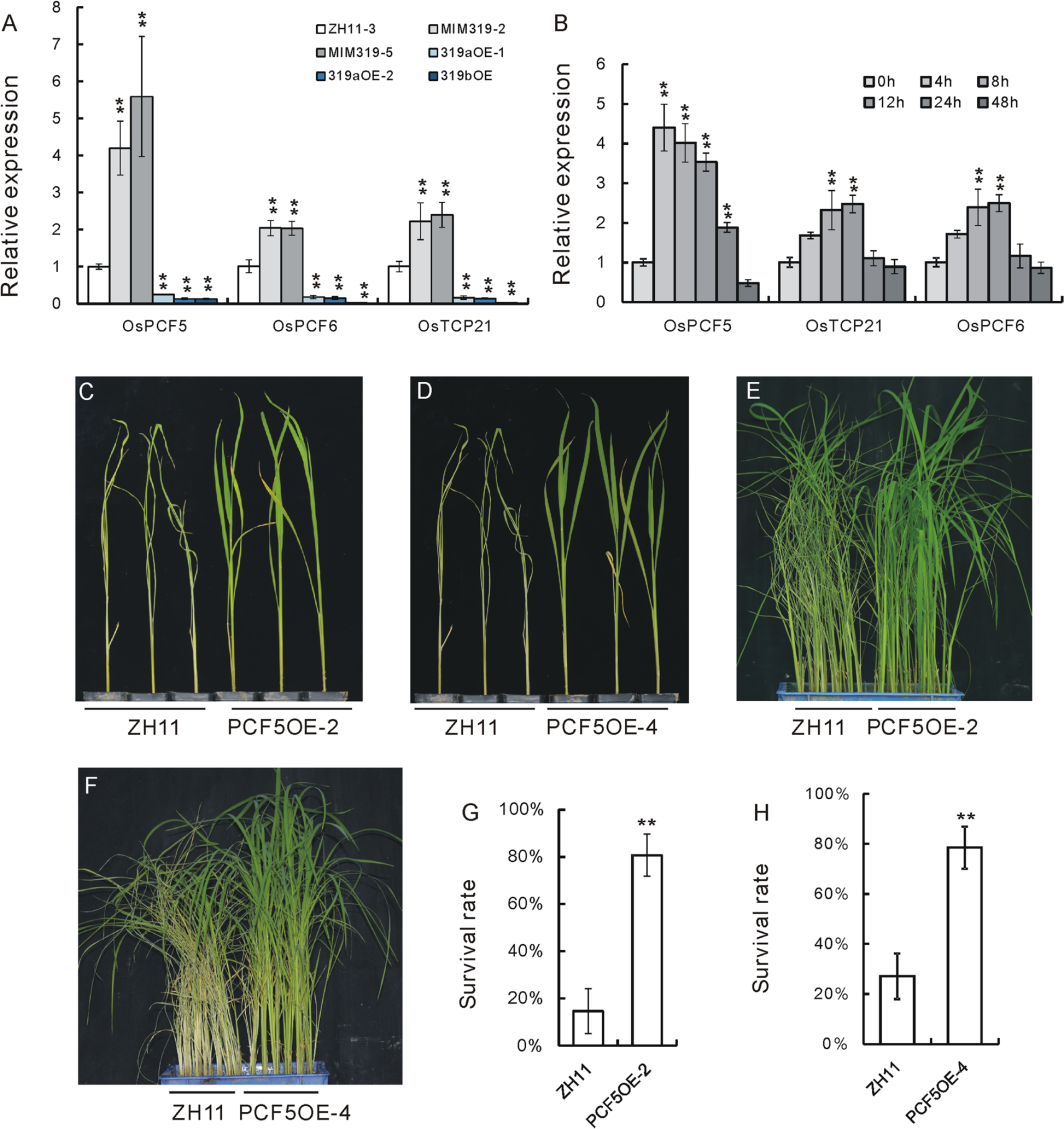
Expression analyses of putative target genes of OsmiR319, functional analysis of the *OsPCF5* gene, and characterization of the OsPCF5 protein. **A** Expressions of *OsPCF5*, *OsPCF6*, and *OsTCP21* in miR319aOE lines, MIM319OE lines, and ZH11 plants as revealed by qRT-PCR (*n* = 3). The expression level in ZH11 was set as 1.0, and asterisks indicate significant differences compared with ZH11 plants as determined by Student’s *t* test (**, *P* < 0.01; *, *P* < 0.05). Individual data values are provided in table S8. **B** Expressions of *OsPCF5*, *OsPCF6*, and *OsTCP21* upon BPH infestation as revealed by qRT-PCR (*n* = 3). The expression level at 0 h was set as 1.0, and asterisks indicate significant differences comparing with 0 h as determined by Student’s *t* test (**, *P* < 0.01). Individual data values are provided in table S9. **C**,**D** Individual tests of PCF5OE-2, PCF5OE-4 plants, and ZH11. **E**,** F** Small population tests of PCF5OE-2 and PCF5OE-4 plants after BPH infestation compared with ZH11. **G**, **H** Survival rates of the plants after BPH feeding in **E** and **F** respectively. Asterisks in **G** and **H** indicate significant differences compared with ZH11 as determined by Student’s *t* test (**, *P* < 0.01; *, *P* < 0.05). Individual data values are provided in table S11 and S12 respectively

The predicted target genes of OsmiR319 are TCP-encoding genes (http://structuralbiology.cau.edu.cn/PNRD). To verify that OsmiR319 represses these target genes, we first measured their expressions in miR319bOET plants and the WT, TP309. It was revealed that among all the putative target genes, *OsPCF5* (LOC_Os01g11550), *OsPCF6* (LOC_Os03g57190), and *OsTCP21* (LOC_Os07g05720) were obviously downregulated in miR319bOET plants (Additional files 1F and 5). For further verification, we measured the expressions of *OsPCF5*, *OsPCF6*, and *OsTCP21* in miR319aOE, miR319bOE, MIM319OE, and WT ZH11 plants. All three genes were downregulated in miR319aOE and miR319bOE plants while upregulated in MIM319OE plants (Fig. 2A, Additional file 1G), thus suggesting the repressive regulation of OsmiR319 on these three target genes.

To further identify target genes potentially involved in BPH response, we measured the expressions of *OsPCF5*, *OsPCF6*, and *OsTCP21* genes in response to BPH infestation. We found that all three genes were induced by BPH infestation, with *OsPCF5* showing the highest degree of induction (Fig. 2B, Additional file 1H). We selected *OsPCF5* for further functional verification by genetic overexpression. The resulting PCF5OE transgenic plants had greatly increased levels of *OsPCF5* mRNA transcripts (Additional files 1I and 6). We then chose lines PCF5OE-2 and PCF5OE-4, which showed the highest expression of *OsPCF5* and tested their individual responses to BPH infestation. The PCF5OE plants died later than the WT (Figs. 2C, D). Next, we used small population assay to check the BPH resistance of PCF5OE-2 and PCF5OE-4 plants. Also, the two lines of PCF5OE plants died later than the WT (Fig. 2E, F), line PCF5OE-2 showed a plant survival rate of 81%, while the corresponding WT was 15% (Fig. 2G, Additional file 1J), and PCF5OE-4 showed a plant survival rate of 78.5% when the corresponding WT was 27.1% (Fig. 2H, Additional file 1K). Therefore, *OsPCF5* positively regulates BPH resistance as the target gene of OsmiR319.

### Interaction of OsPCF5 with several MYB proteins

OsPCF5 is a TCP domain-containing protein that belongs to the bHLH family (Additional file 7). Given that bHLH proteins usually form MBW complexes to fulfill their function, we looked for MYB proteins able to interact with OsPCF5 to mediate rice response to BPHs.

**Fig. 3:**
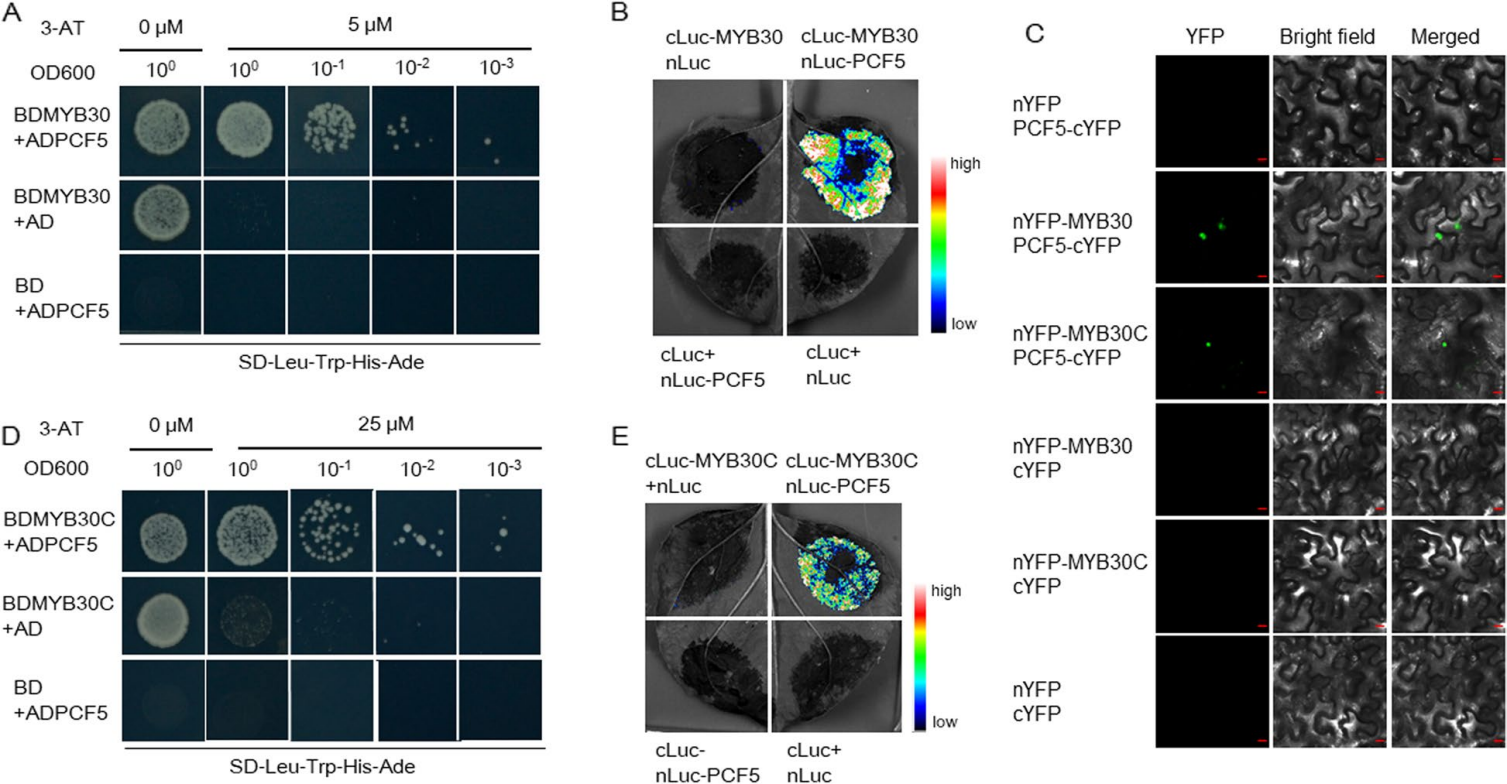
Interaction of OsPCF5 with OsMYB30 and OsMYB30C in vitro and in vivo. **A**, **D** Y2H assay showing OsPCF5 can interact with OsMYB30 and OsMYB30C in yeast cells, respectively. Transformed yeast cells were 1-, 10-, 100-, and 1000-fold diluted and spotted on SD-Leu-Trp-His-Ade medium containing 5 mM 3-amino-1, 2, 4-triazole (3-AT). The empty vectors were used as controls. B,E Interactions of OsPCF5 with OsMYB30 and OsMYB30C as shown by a LCA on the same *N. benthamiana* leaf, respectively. **C** BIFC assay to verify the interaction of OsPCF5 with OsMYB30 and OsMYB30C on *N. benthamiana* leaves, respectively. Bars = 10 μm

OsMYB30 has been reported to positively regulate *OsPAL6* and *OsPAL8* genes, which in turn mediate BPH resistance by upregulating lignin biosynthesis [39]. We first investigated the ability of OsPCF5 to interact with OsMYB30. A yeast two-hybrid assay (Y2H) revealed that yeast cells harboring both OsPCF5 and OsMYB30 could grow on medium lacking Ade, His, Leu, and Trp amino acids (Fig. 3A). This interaction was further verified by a luciferase complementation assay (LCA; Fig. 3B) and a bimolecular fluorescence complementation (BIFC) assay (Fig. 3C).

According to our previous research, OsmiR319 regulates rice cold tolerance [20], and another MYB protein–encoding gene, *OsMYB30C*, also has a role in cold tolerance [40]. We therefore investigated whether OsMYB30C can also interact with OsPCF5. In an Y2H assay, yeast cells harboring both OsPCF5 and OsMYB30C were able to grow on medium lacking Ade, His, Leu, and Trp (Fig. 3D). This interaction was further confirmed by a LCA (Fig. 3E) and a BIFC assay (Fig. 3C). According to these results, OsPCF5 can interact not only with OsMYB30, but also with OsMYB30C.

*MYB* genes function extensively in plant development and metabolic regulation [34]. To detect whether MYB proteins participate in BPH resistance, we carried out an RNA-seq analysis of ZH11 and resistant variety RHT subjected to BPH feeding, which uncovered numerous *MYB* genes responsive to BPH infestation (Additional files 1L and 8). Consequently, *MYB* genes may have active roles in BPH resistance.

**Fig. 4:**
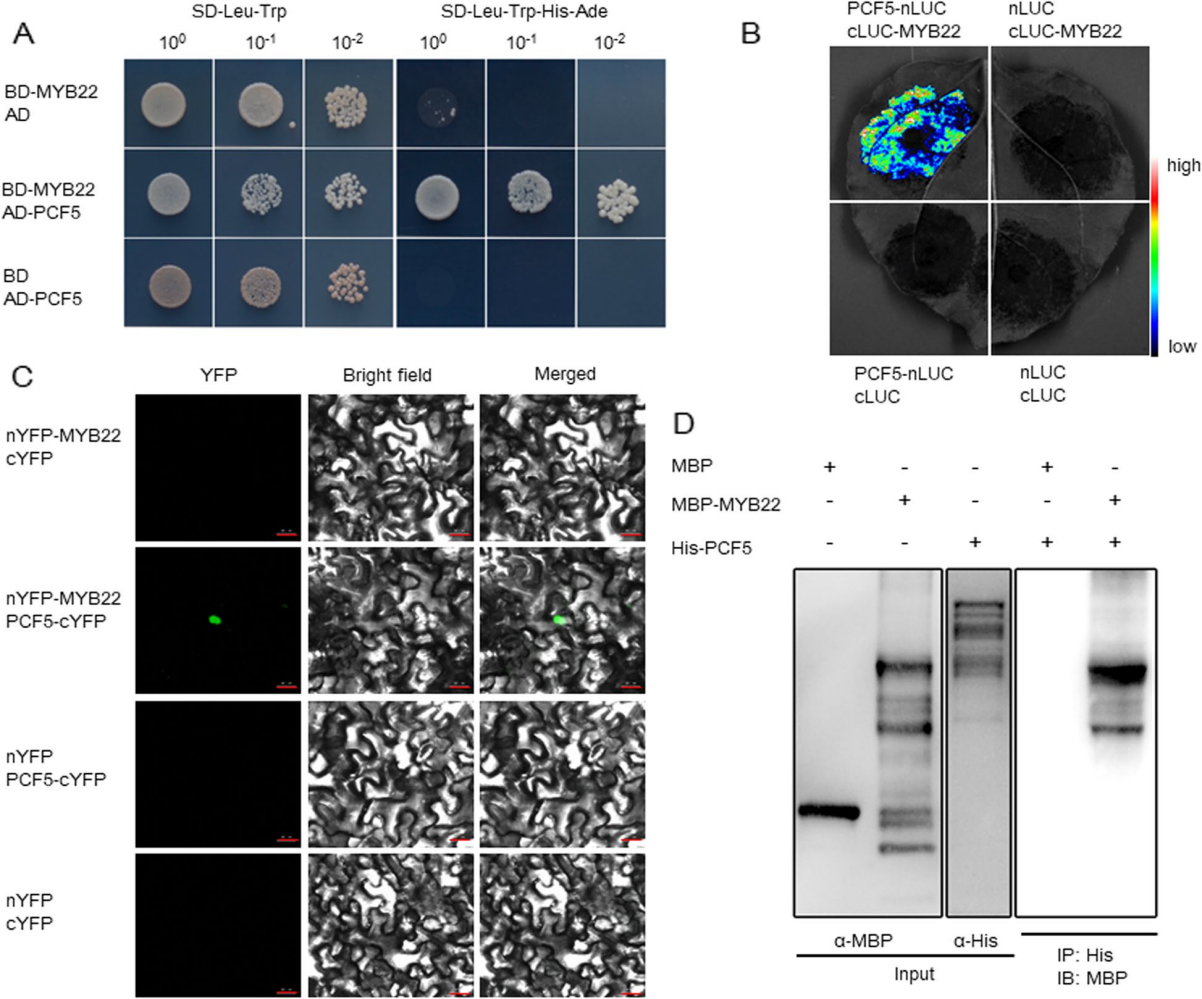
Interaction of OsPCF5 with OsMYB22 in vitro and in vivo. **A** Y2H assay showing OsPCF5 can interact with OsMYB22 in yeast cells. Transformed yeasts were 1-, 10-, 100-, and 1000-fold diluted and spotted on SD-Leu-Trp-His-Ade medium. The empty vectors were used as controls. **B** Interactions of OsPCF5 with OsMYB22 as shown by a LCA on the same *N. benthamiana* leaf. **C** BIFC assay to verify the interaction between OsPCF5 and OsMYB22 in *N. benthamiana* leaves. Bars = 20 μm. **D** Pull-down assay to verify the interaction between OsPCF5 and OsMYB22

Also, we screened our mutant population [43] for BPH resistance and found a resistant mutant with a T-DNA insertion in the *OsMYB22* gene promoter (data not shown). To explore whether *OsMYB22* is also involved in BPH resistance, we first checked whether OsMYB22 can interact with OsPCF5 before carrying out a functional verification. In a Y2H assay, cells harboring both OsPCF5 and OsMYB22 grew well on medium lacking Leu, Trp, Ade, and His amino acids (Fig. 4A), thus indicating the interaction of these two proteins in yeast cells. In a LCA, the combination of OsPCF5 and OsMYB22 produced strong florescence (Fig. 4B). This interaction was further verified by BIFC (Fig. 4C). Finally, in a pull-down assay His-tagged OsPCF5 was able to bind with MBP-tagged OsMYB22, but not with MBP alone (Fig. 4D). Therefore, we identified another MYB protein, OsMYB22 that interacts with OsPCF5.

### Genetic function of OsMYB22 and its genetic relation with OsmiR319b in BPH resistance

**Fig. 5:**
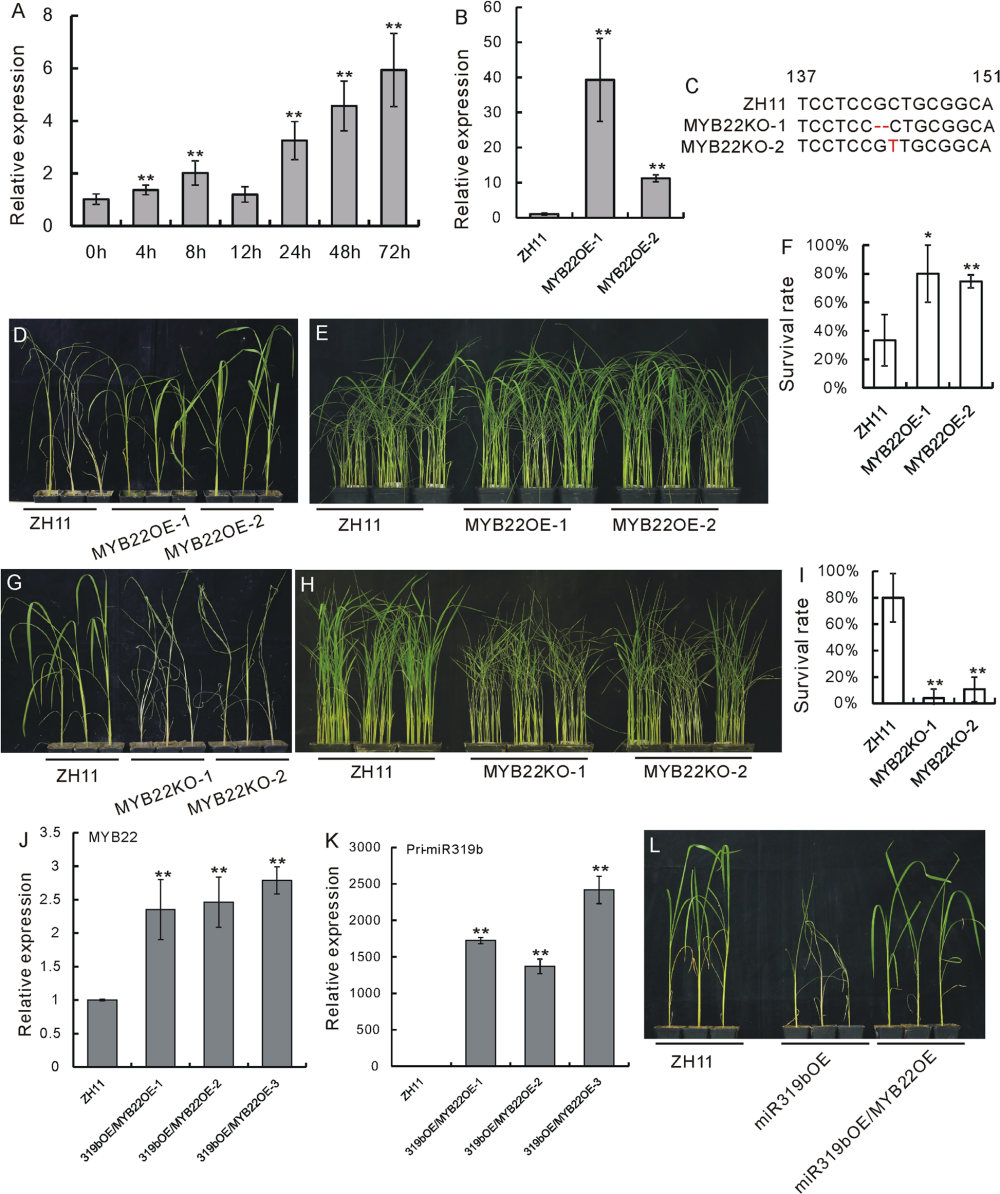
Expression and functional analyses of the *OsMYB22* gene in response to BPHs. **A** Expression of *OsMYB22* after BPH infestation as revealed by qRT-PCR (*n* = 3). The expression level at 0 h was set as 1.0, and asterisks indicate significant differences comparing with 0 h as determined by Student’s *t* test (**, *P* < 0.01). Both Ubiquitin and Actin genes were used as references in this result. Individual data values are provided in table S14. **B** Expression of the *OsMYB22* gene in MYB22OE lines and WT plants as revealed by qRT-PCR (*n* = 3). The expression level in ZH11 was set as 1.0, and asterisks indicate significant differences compared with ZH11 plants as determined by Student’s *t* test (**, *P* < 0.01). Individual data values are provided in table S15. **C** Sketch map indicating the sequences of DNA edited in MYB22KO lines, MYB22KO-1, and MYB22KO-2. **D** Individual tests of MYB22OE-1 and MYB22OE-2 lines in comparison with ZH11. **E** Small population assays of MYB22OE-1 and MYB22OE-2 lines in comparison with ZH11. **F** Statistical analysis of the survival rates of plants in the small population assay in **E**. Asterisks indicate significant differences compared with ZH11 plants as determined by Student’s *t* test (**, *P* < 0.01). Individual data values are provided in table S16. **G** Individual tests of MYB22KO-1 and MYB22KO-2 lines in comparison with ZH11. **H** Small population assays of MYB22KO-1 and MYB22KO-2 lines in comparison with ZH11. **I** Statistical analysis of the survival rates of plants in the small population assay in **H**. Asterisks indicate significant differences compared with ZH11 plants as determined by Student’s *t* test (**, *P* < 0.01). Individual data values are provided in table S17. **J**,** K** Expression of *OsMYB22* (**J**) and OsmiR319b (**K**) in hybrid plants as revealed by qRT-PCR and miRNA qRT-PCR (*n* = 3), respectively. The expression level in ZH11 was set as 1.0, and asterisks indicate significant differences compared with ZH11 plants as determined by Student’s *t* test (**, *P* < 0.01). Individual data values are provided in table S18. **L** Individual tests of the WT, miR319bOE, and cross plants miR319bOE/MYB22OE

To investigate whether *OsMYB22* functions in BPH resistance, we first measured the expression of *OsMYB22* in response to BPH feeding. Accordingly, *OsMYB22* was obviously induced beginning at 4 h after BPH feeding (Fig. 5A, Additional file 1M). Next, we constructed *OsMYB22*-overexpressing transgenic plants (MYB22OE) and *OsMYB22*-edited plants (MYB22KO) using CRISPR-Cas9 technology. *OsMYB22* was obviously upregulated, but to different degrees, in MYB22OE-1 and MYB22OE-2 lines (Fig. 5B, Additional file 1N). In the *T*_0_ generation of MYB22KO plants, we obtained two homozygous lines, MYB22KO-1 and MYB22KO-2, which harbored a single-nucleotide deletion and a single-nucleotide insertion, respectively (Fig. 5C).

We then tested the response of MYB22OE and MYB22KO lines to BPHs. In an individual test, both MYB22OE-1 line and MYB22OE-2 line died later than their WTs (Fig. 5D); also, in a small population assay, MYB22OE plants died later than the WT as manifested by MYB22OE-1 and MYB22OE-1 lines (Fig. 5E, Additional file 9). Furthermore, the survival rates of both MYB22OE lines were obviously higher than that of the WT (Fig. 5F, Additional file 1O). On the other hand, in an individual test, the MYB22KO-1 and MYB22KO-2 plants died earlier than the WT (Fig. 5G). And in a small population assay, the plants of MYB22KO-1 and MYB22KO-2 lines died earlier than the WT (Fig. 5H, Additional file 9), with the survival rates of the MYB22KO-1 and MYB22KO-2 lines obviously lower than that of the WT (Fig. 5I, Additional file 1P). Therefore, from these functional assays, it is obvious that *OsMYB22* positively regulates BPH resistance.

To further ascertain that OsmiR319 and *OsMYB22* function in the same genetic pathway to mediate BPH resistance, we made genetic crosses between miR319bOE and MYB22OE plants. We confirmed that both OsmiR319b and *OsMYB22* were overexpressed in miR319bOE/MYB22OE plants compared with the WT (Fig. 5J, K, Additional file 1Q). When the WT, miR319bOE, and miR319bOE/MYB22OE plants were tested individually for BPH resistance, the response of miR319bOE/MYB22OE plants was similar to that of the WT, whereas miR319bOE plants were more susceptible (Fig. 5L), indicating that the BPH-susceptible character of the miR391bOE plants was complemented by *OsMYB22* overexpression.

### Genetic function of OsMYB30C and its genetic relation with OsmiR319b in BPH resistance

**Fig. 6:**
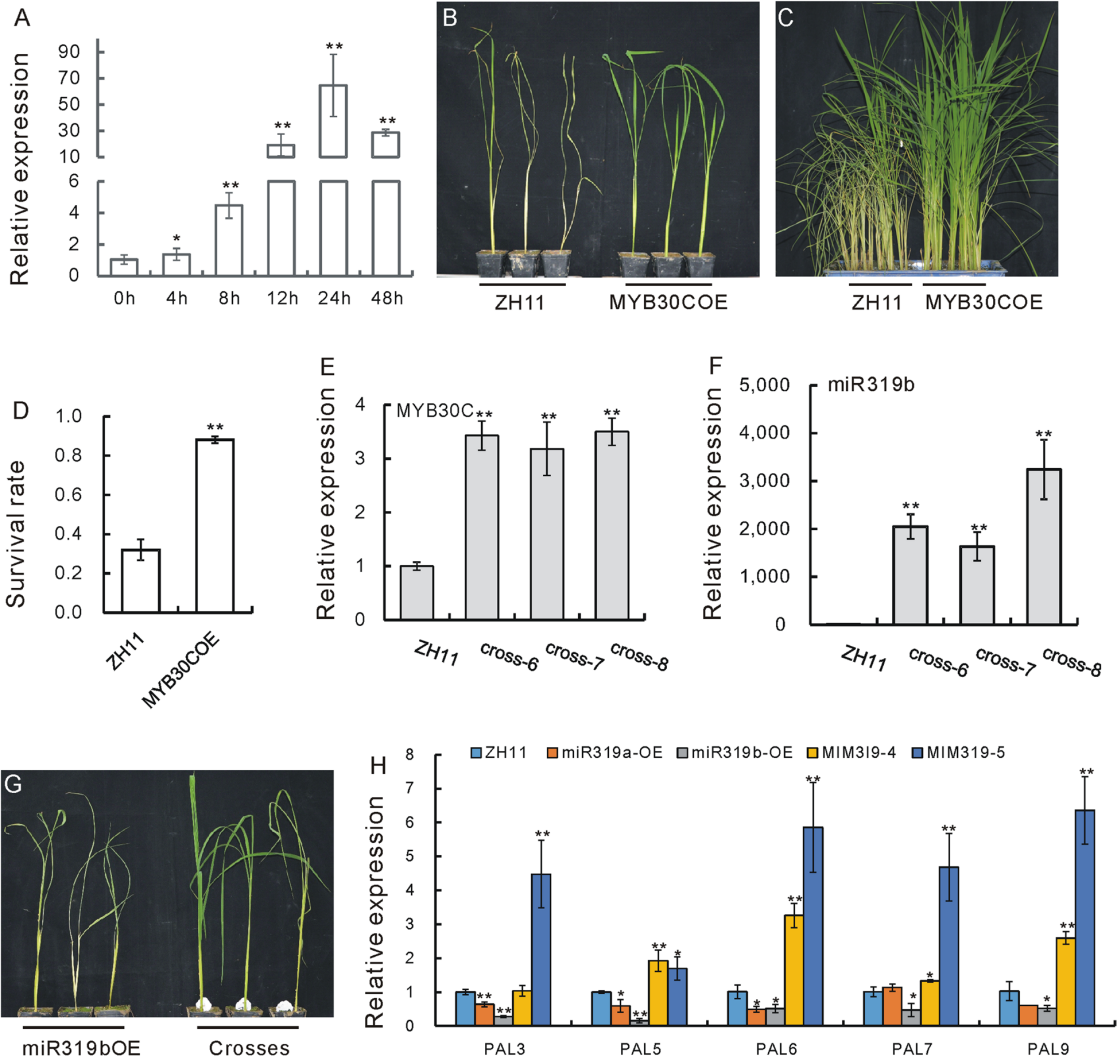
Functional analysis of the *OsMYB30C* gene in response to BPHs. **A** Expression profile of *OsMYB30C* after BPH infestation as revealed by qRT-PCR (*n* = 3). The expression level at 0 h was set as 1.0, and asterisks indicate significant differences compared with 0 h as determined by Student’s *t* test (**, *P* < 0.01; *, *P* < 0.05). Both Ubiquitin and Actin genes were used as references in this result. Individual data values are provided in table S19. **B** Individual tests of MYB30COE and WT ZH11 plants. **C** Picture of small population assays of MYB30COE and WT ZH11 plants. **D** Statistical analysis of the survival rates of plants in the small population assay in **C**. Asterisks indicate significant differences compared with that of ZH11 as determined by Student’s *t* test (**, *P* < 0.01). Individual data values are provided in table S20. **E**,** F** Expression of *OsMYB30C* (**E**) and OsmiR319b (**F**) in hybrid plants as revealed by qRT-PCR and miRNA qRT-PCR (*n* = 3), respectively. The expression level in ZH11 was set as 1.0, and asterisks indicate significant differences compared with ZH11 plants as determined by Student’s *t* test (**, *P* < 0.01). Individual data values are provided in table S21 and S22. **G** Individual tests of WT, miR319bOE, and three hybrid miR319bOE/MYB30COE plants against BPH infestation. **H** Expression of several *OsPAL* genes in the miR319aOE, miR319bOE, MIM319OE and ZH11 plants as revealed by qRT-PCR (*n* = 3). The expression level in ZH11 was set as 1.0, and asterisks indicate significant differences compared with ZH11 plants as determined by Student’s *t* test (**, *P* < 0.01; *, *P* < 0.05). Individual data values are provided in table S23

Next, we wondered if the *OsMYB30C* gene also functions in BPH response. An expression analysis revealed that *OsMYB30C* is obviously induced by BPH feeding (Fig. 6A, Additional file 1R). When we evaluated the response of MYB30COE plants to BPHs either individually or in a small population assay, MYB30COE plants died later than the WT in both cases (Fig. 6B, C). And in a small population test, the survival rate of the MYB30COE plants was much higher than that of ZH11 (Fig. 6D, Additional file 1S). These results indicate that OsMYB30C positively regulates resistance to BPHs.

To investigate whether *OsMYB30C* functions in the same genetic pathway with *OsPCF5* in response to BPHs, we crossed MYB30COE plants with miR319bOE plants. Both *OsMYB30C* and OsmiR319b were overexpressed in the resulting hybrid plants (Fig. 6E, F, Additional file 1T, U). Individual miR319bOE/MYB30COE plants subjected to BPH feeding died later than the female parent, miR319bOE (Fig. 6G), which indicates that the susceptibility of the miR319bOE plants was complemented by the resistance of the MYB30COE plants.

Given that *OsMYB30* positively regulates *OsPAL6* and *OsPAL8* to modulate BPH [39] resistance and that OsPCF5 interacts with several MYB proteins, including OsMYB30, we wondered whether *OsPAL* genes were also influenced by the OsmiR319/OsPCF5/MYB complex. Therefore, the expressions of several *OsPAL* genes in miR319aOE, miR319bOE, MIM319OE, and ZH11 plants were detected, we found that these genes were generally downregulated in miR319aOE and miR319bOE plants and upregulated in MIM319OE ones (Fig. 6H, Additional file 1V). This result suggests that the OsmiR319/OsPCF5 module might mediate BPH resistance by influencing *OsPAL* genes.

## Discussion

The functions of miRNAs in mediating plant and environmental interactions are being increasingly revealed [13]. However, relatively few miRNAs have been studied in regard to their function in mediating BPH resistance, the most notorious insect pest to rice plantation. Several studies have indicated the involvement of miRNA in BPH resistance at whole genome level [14–16]. We have previously shown that OsmiR396s negatively control BPH resistance through the flavonoid synthesis pathway, with the target OsGRF8 direct regulating on the flavonoid synthesis gene *OsF3H* [16]. Also, OsmiR156 is a negative regulator to BPH resistance, which might function through the JA biosynthesis pathway [17]. Here, we studied the role of OsmiR319 in response to BPHs and found that OsmiR319 overexpression greatly enhanced the susceptibility of rice plants to BPHs (Fig. 1G, H), whereas downregulation of OsmiR319 through the mimicry technology increased BPH resistance (Fig. 1J, K). Thus, OsmiR319 negatively regulated BPH resistance, this character was repeatedly verified in several different genetic backgrounds of rice (Additional file 4). Meanwhile, overexpression of one target gene, *OsPCF5*, was proved to be resistant to BPH (Fig. 2C–H). Besides, other target genes of OsmiR319, such as *OsPCF6* and *OsTCP21*, might also mediate BPH resistance, as their rapid, active response to BPH infestation is similar to that of *OsPCF5* (Fig. 2B). Therefore, OsmiR319 together with at least one of its target genes, *OsPCF5*, were proven to mediate resistance to BPHs.

As one kind of the most conserved miRNAs, miR319 has been identified in various plant species from mosses to flowering plants [44]. Meanwhile, miR319 functions in various aspects of plant development and physiology, such as leaf morphogenesis and differentiation [19, 22], organ curvature, hormone biosynthesis [26, 30, 45, 46], fiber cell elongation in cotton [47] and trichome development in *Populus tomentosa* [30], cold tolerance in rice [20], salt tolerance, and ethylene synthesis in switchgrass [25]. Recent studies find that miR319 regulates plant architecture and grain yield in common wheat [48], and tillering and grain yield in rice [27]. Although the increased trichome density in miR319 overexpression plants accounts for the promoted insect resistance in *Populus tomentosa* [30], similar characteristics in rice overexpressing OsmiR319 was not found (data not shown), so that the insect resistance character of MIM319 might not be attributed to trichome development. There should be other mechanism mediating the resistance of mi319 to BPH. Meanwhile, it is revealed that OsmiR319 may function by promoting cell proliferation while inhibiting an increase in cell numbers through negative regulation of its TCP targets [21]. This proliferation-promoting characteristic may cause rice plants overexpressing OsmiR319 not only be more vulnerable to some insect pests such as BPHs, as demonstrated in this study, but also have increased susceptibility to some fungal pathogens and viruses [28, 29]. Moreover, miR319-targeted *TCP4* promotes secondary cell wall formation and programmed cell death in *Arabidopsis* [49]. If conserved in rice, the proliferation-promoting characteristic of miR319 might be one mechanism through which miR319OE plants increased susceptibility to BPHs, while MIM319 plants and PCF5OE plants increased resistance to BPHs. Furthermore, miR319 positively regulates insect resistance in *Populus tomentosa*, while negatively regulated BPH resistance in rice, this difference in function of miR319 (positive or negative) might further indicates difference in downstream signaling pathways, which needs further study, especially under the circumstance that miRNA is multi-functional.

For further mechanism detection of the miR319/PCF5-mediated BPH resistance, we carried our series biochemical assays and revealed that OsPCF5 can interact with several MYB proteins, such as OsMYB30, MYB30C, and MYB22 (Figs. 3 and 4). OsMYB30 has been proved to positively regulate rice resistance to BPHs through direct trans-activation of *OsPAL6* and *OsPAL8* genes, which in turn positively regulate rice BPH resistance by controlling the biosynthesis and accumulation of SA and lignin [39]. OsMYB30C has previously been proved to positively regulate rice resistance to fungal pathogens through enhanced lignification [41]. Consistent with this function, we showed that OsMYB30C positively regulates rice resistance to BPHs (Fig. 6B, C), thus adding further evidence for the involvement of OsMYB30C in rice immunity regulation. Meanwhile, we identified a new member of the MYB family, OsMYB22, as a positive regulator of rice resistance to BPHs (Fig. 5D–I), although it might function through negatively regulating the flavonoid synthesis gene *OsF3’H*, which negatively regulates BPH resistance and positively regulates rice blast disease resistance [50, 51]. Both MYB30COE and MYB22OE could complement the susceptible characteristic of miR319bOE plants (Figs. 5L and 6G), indicating that OsmiR319 and these *MYB* genes function in the same genetic pathway to mediate resistance to BPHs. Increasing evidence points to the regulatory involvement of MYB proteins in phenylpropanoid metabolism [34]. In particular, OsMYB30 has been previously proven to directly control *OsPAL6* and *OsPAL8* in the phenylpropanoid synthetic pathway to regulate BPH resistance [39]. In the present study, we provided further evidence that OsMYB30 can interact with OsPCF5 (Fig. 3A–C). We proposed that these newly identified OsPCF5-interacting MYB proteins might also regulate BPH resistance through the phenylpropanoid pathway, as *OsPAL* genes were generally upregulated in MIM319OE plants while downregulated in miR319OE plants (Fig. 6H).

JA synthesis and signaling pathway is also extensively involved in BPH resistance. Generally, JA might function positively against BPH infestation, with lines of deficiency in *AOC* gene, the gene functioning in JA synthesis, and *MYC2* gene, which functions in JA signaling, which were both susceptible to BPH [52]. The *OsLOX11* gene positively regulates BPH resistance [53], while *OsLOX9* gene negatively regulates BPH resistance and might mediate the crosstalk between the JA- and ethylene pathway [54, 55]. Several studies revealed the tight association between miR319 and JA synthetic and signaling pathways in immunity. In tomato, it is suggested that miR319 serves as a systemic signal responder and regulator that modulated the root-knot nematode systemic defensive response mediated by JA [56]. Rice blast disease induced the expression of OsmiR319, and OsmiR319/OsTCP21 positively regulate disease resistance through the JA biosynthesis and signaling pathway [28]. Infection of rice ragged stunt virus (RRSV) induced expression of OsmiR319, which suppressed JA-mediated defense to facilitate virus infection and symptom development [29]. RRSV is among the virus that could be transmitted by BPHs; therefore, it is consistent that OsmiR319 overexpression is susceptible to both RRSV and BPHs. Whether JA pathway functions in the OsmiR319 mediated resistance to BPH still needs further investigation.

**Fig. 7:**
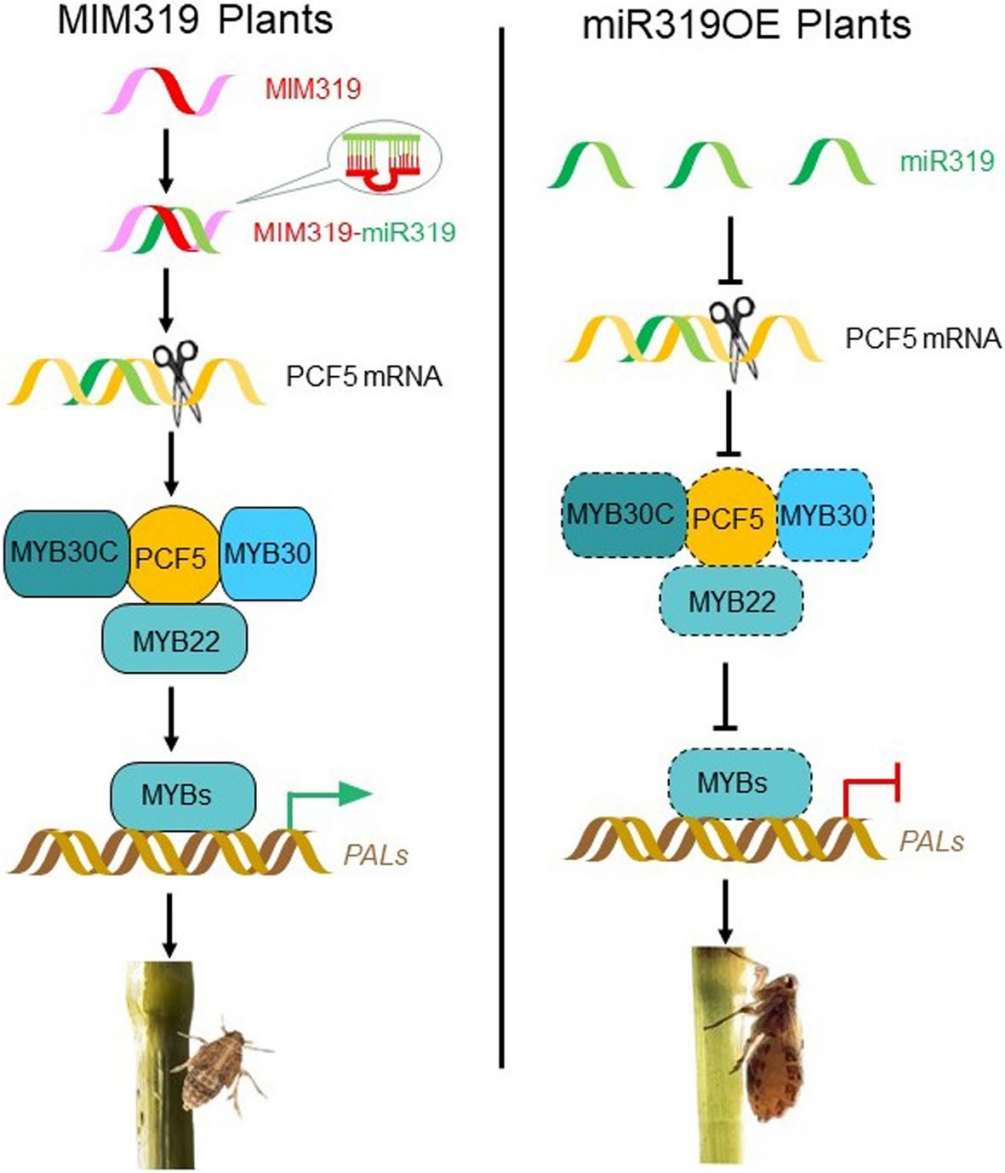
Schematic representation of the mechanism of the OsmiR319-OsPCF5-OsMYB pathway in BPH resistance. In MIM319OE plants, the production of MIM319 inhibited miR319, and thus elevated the amount OsPCF5 protein, along with the association of OsPCF5 with other OsMYB proteins, therefore the activation of MYB proteins on *PAL* genes were enhanced and thus promoted BPH resistance. In miR319OE plants, the sufficient amount of miR319 inhibited *OsPCF5* gene, and therefore reduced OsPCF5 proteins and influenced its association with other MYB proteins, thus the activation of *PAL* genes was impacted, which made the plants more vulnerable to BPH

Taken together, our results indicated that BPH infestation enhances expression of both OsmiR319 and its target genes; to elucidate the possible resistance mechanism mediated by OsmiR319, we performed molecular, genetic, and biochemical analysis and described the involvement of an OsmiR319-OsPCF5-OsMYB pathway in BPH response. We proved that OsmiR319 negatively regulated BPH resistance. In MIM319OE plants, the production of MIM319 inhibited miR319, and thus elevated the amount OsPCF5 protein, along with the association of OsPCF5 with other OsMYB proteins; therefore, the activation of MYB proteins on *PAL* genes were enhanced and thus promoted BPH resistance. However, in miR319OE plants, the sufficient amount of miR319 inhibited *OsPCF5* gene, and therefore reduced OsPCF5 proteins and influenced its association with other MYB proteins, thus the activation of *PAL* genes was impacted, which made the plants more vulnerable to BPH (Fig. 7).

## Conclusions

We identified that OsmiR319/OsPCF5 module regulated BPH resistance in rice. OsmiR319 negatively regulated BPH resistance, while OsPCF5 positively regulated BPH resistance. OsPCF5 function through tight association with OsMYB22, OsMYB30, and OsMYB30C, three positive regulators of BPH resistance. Genetic relation between OsmiR319 and OsMYB22, OsmiR319 and OsMYB30C further proved the function of OsmiR319-OsPCF5-OsMYB genetic pathway in BPH resistance. And the phenylpropanoid pathways might function downstream of the OsmiR319-OsPCF5-OsMYB genetic pathway to mediate BPH resistance.

## Methods

### Plant materials and BPH population

The wild type (WT) rice plants used in this study were varieties ZH11 (*Oryza sativa* L. subsp. *japonica* cv. Zhonghua No.11, ZH11), TP309 (*Oryza sativa* L. subsp. *japonica* cv. TaiPei 309, TP309), Kasalath (*Oryza sativa* L. subsp. *indica* cv. Kasalath), TN1 (*Oryza sativa* L. subsp. *indica* cv. Taichung Native 1, TN1), and RHT (*Oryza sativa* L. subsp. *indica* cv. Rathu Heenati, RHT). All rice plants were cultivated under field conditions at two different experimental stations in Shanghai (30° N, 121° E) and Lingshui (Hainan Province, 18° N, 110° E), China. Rice seedlings were cultured in the phytotron in CAS Center for Excellence in Molecular Plant Sciences, with 30/24 ± 1℃ day/night temperature and 50–70% relative humidity, and a light/dark period of 14 h/10 h was used to culture rice seedlings.

The BPH population was originally obtained from rice fields in Shanghai, China, and maintained on susceptible rice variety TN1 in a climate-controlled room at 26 ± 2 °C, 12 h/12 h light/dark cycle, and 80% relative humidity.

### Plasmid construction and plant transformation

For *OsMIR319a* and *OsMIR319b* overexpression, fragments containing respective stem-loop structures of pri-OsmiR319a (286 bp) and pri-OsmiR319b (362 bp) respectively cloned into p130135SNOS vector using *BamH*I and *Kpn*I double digestion. Construction of vector overexpressing OsmiR319 target mimic (MIM319) was carried out according to previous reports [57, 58]. Briefly, four nucleotide “TAGA” was inserted into the 10–11 sites of the sequence of OsmiR319, and substituted the original miR399 targeting sequence in the non-coding *IPS1* gene using overlapping PCR method. Then the modified MIM319-IPS1 cassette was cloned into the p130135SNOS vector through *Bam*HI and *Sac*I double digestion. The primer sequences for *OsMIR319a* and *OsMIR319b* overexpression and those specific for MIM319-IPS1 amplification, MIM319F, and MIM319R are listed in Additional file 1W.

For *OsPCF5* and *OsMYB22* overexpression, the full-length cDNA of them were respectively amplified and cloned into p130135SNOS vector respectively through double digestion or fusion using ultra one step cloning kit (Vazyme). The enzymes used for *OsMYB22* overexpression were *BamH*I and *Kpn*I, and those for *OsPCF5* overexpression were *BamH*I and *Spe*I.

For knocking out of *OsMYB22*, guider DNA (gDNA) was synthesized and cloned into the pOs-sgRNA vector, and then transferred to the pH-Ubi-cas9-7 vector through LR reaction. gDNA used for *OsMYB22* is listed in Additional file 1W.

Plasmids were respectively transformed into ZH11, Kasalath, or TP309 as needed through *Agrobacterium*-mediated genetic transformation in Towin Biotechnology Company (www.towinbio.com/).

### BPH resistance detection and measurements

Individual test assay was carried out at seedling stage using at least six replicates of each cultivar or line as previously described [9, 59]. Each seedling about 3-week stage was infested with 12-s instar BPH nymphs. Plant status were checked daily, and 6–9 days later, the plants were scored as susceptible (dead) or resistant (alive) and pictures were taken.

For small population assay, about 50 plants of tested lines and the control lines were planted in a plate in the mud for 1 month till the third-leaf stage, and fed to BPH population in appropriately 10–15 first-instar nymphs per plant, and the plant status (alive or dead) were surveyed daily in the following week. At around 7–12 days after infestation, when there is obvious difference in the status of the plants, the survival rates of the tested plants and the control plants were counted with at least three biological repeats. And the data were statistically analyzed. Data was shown as mean ± SD. Also, another substitution for small population was used. Twenty-five plants of tested line and control line were each planted in a small pot with mud for about 1 month till the third-leaf stage, with at least three repeats. Then pots of tested line and control line were put in parallel and fed to BPH population in appropriately 5–8 s instar nymphs per plant, and the plant status (alive or dead) were surveyed daily in the following week. Pictures of the plant status were taken before BPH infestation and when either of the tested line or the control line were nearly dead at around 7–12 days after infestation, and the survival rates of the plants were surveyed simultaneously using at least three repeats and statistically analyzed. Data was shown as mean ± SD.

For survival rate analysis of the BPHs, 35 first-instar nymphs were placed on each 6-week-old plant and covered with a cylindrical Mylar cage. The number of the BPHs was counted daily in the following 10 days. Experiments were carried out with at least three repeats, and the survival rates were statistically analyzed. Data was shown as mean ± SD.

For the bulked seedling test, about 20 seeds of each line were sown in a row of 15 cm length in a plastic box. TN1 or Kasalath were randomly planted as susceptible controls. At the third-leaf stage, the seedlings were infested with second to third instar nymphs of BPH at eight insects per seedling. When all of the seedlings of susceptible control died, the plants were examined and each seedling was given a score of 0, 1, 3, 5, 7, and 9 according to Huang et al. [60]. At least three repeats were carried out and the scores of the tested plants were statistically counted. Data was shown as mean ± SD.

### Phenotyping of plants and leave characteristics

Plant materials (leaves and whole plants) were photographed using a Canon EOS7D digital camera. For statistical analysis, leaf length and width of 15 top-leaves at mature stage were measured and statically analyzed. Data was shown as mean ± SD.

### RNA isolation and quantitative real-time RT-PCR (qRT-PCR) analysis

For OsmiR319 verification in the miR319aOE, miR319bOE, and MIM319 lines, and OsmiR319 target gene expression, and gene expression in various signaling pathway, leaves of the seedlings were used for RNA extraction. Total RNAs were extracted using TRIzol (Yeasen) following the manufacturer’s instructions, then 2 μg total RNA was reverse transcribed using the First Strand cDNA Synthesis Kit (Toyobo) according to the manufacturers’ instructions. qRT-PCR was performed with the SYBR Green Real-time PCR Master Mix Kit (Toyobo) with a reaction system containing 10.0 μl of 2 × SYBR Green Real-time PCR Master Mix, 0.4 μl of each primers, 4 μl of 10 ng/μl cDNA, and 5.2 μl of ddH_2_O. qRT-PCR was conducted using an Eppendorf realplex^2^ System. Each sample was analyzed in triplicate, and the mean values of the technical replicates were recorded for each biological replicate. The rice *Actin* (LOC_Os03g50885) and *Ubiquitin* (LOC_Os01g22490) were used as reference genes to normalize expression levels. Relative gene expression in different individuals was analyzed using the 2^−ΔΔCT^ method with *Actin* [61], compared with wild type. Methods for analyzing qPCR with two reference genes were according to the website (https://toptipbio.com/qpcr-multiple-reference-genes/). The average value of the biological repetitions of the BPH feeding treatment at 0 h was used as the calibrator to calculate the ΔCT of each sample under the specific primers of each gene, and the relative quantity (RQ) of each gene in each sample was calculated with 2^ΔCT^. The relative expression of gene of interest (GOI) at different time points was calculated by RQ_GOI_/Geomean (RQ_actin_, RQ_Ubiquitin_).

For gene expression analysis responsive to BPHs, 3-week-old rice seedlings were individually infested with 12-s instar BPH nymphs that had been starved for 2 h; before infestation, leaf sheaths were collected and marked as 0 h, and then leaf sheaths were collected after BPH infestation for 2, 4, 8, 12, and 24 h for RNA extraction and the following reverse transcription and qRT-PCR.

All the primer sequences used in vector construction, qRT-PCR, and other analysis in this study are listed in Supplementary table S1.

### Yeast two-hybrid (Y2H) assay

The open reading frame (ORF) of *OsPCF5* and the *MYB* genes were respectively amplified and cloned into the gateway cloning vector pCR8/GW/TOPO (Invitrogen), and transferred to the prey vector pGAD-T7 and bait vector pGBK-T7 respectively through LR reaction using Gateway LR Clonase II (Invitrogen). The prey and bait vectors plasmids were co-transformed into yeast strain AH109 and grew on SD-Leu-Trp solid media (Clontech), and then the strains were transferred to the SD-Ade-His-Leu-Trp solid media to test the interaction between the prey and the bait.

### Luciferase complementation assay (LCA)

LCA was carried out basically as described [62]. The full-length cDNAs of *OsMYB30*, *OsMYB30C*, *OsMYB22*, and *OsPCF5* were amplified and respectively cloned into pCAMBIA1300-NLuc and pCAMBIA1300-CLuc vectors. All the constructs were transformed into *A. tumefaciens* GV3101. Agrobacterium cells were re-suspended in infection solution (10 mM MES, 10 mM MgCl^2^, and 200 μM acetosyringone) at OD600 = 1.0. The prepared suspensions were infiltrated into healthy *Nicotiana* *benthamiana* (*N.* *benthamiana*) leaves which have grown for about 1 month using a 1-ml needless syringe. After light avoidance for one night, the plants were put under normal growth condition. At 48 h post-infiltration the LUCfluorescent signals were examined by a CCD camera 48 h later.

### Bimolecular fluorescence complementation (BiFC) assay

For the BiFC assay, full-length cDNAs of *OsMYB30*, *OsMYB30C*, *OsMYB22*, and *OsPCF5* were amplified and cloned into pXY106 and pXY104 vectors to generate nYFP-OsMYBs and OsPCF5-cYFP, respectively. The recombinant vectors were transformed into *A. tumefaciens* GV3101. After centrifugation, the bacteria were collected and suspended in infection solution (10 mM MES, 10 mM MgCl_2_, and 200 μM acetosyringone) and infiltrated into *N. benthamiana* leaves. Forty-eight hours later, fluorescent signals were monitored using a laser confocal scanning microscope (LSM 880, Zeiss).

### Pull-down assay

For in vitro pull-down assay, the full-length cDNAs of *OsMYB22* and *OsPCF5* were amplified and cloned into the pMAL-C5X or pCOLD-TF vectors respectively, and transformed into *E.coli* strain BL21 to express maltose-binding protein (MBP)–tagged OsMYB22 (MBP-OsMYB22) and His-TF-tagged OsPCF5 (His-OsPCF5). Appropriate His-OsPCF5 and MBP-OsMYB22 or MBP was incubated with Ni–NTA Resin (L00250-50) at 4 °C for 2 h in 200 μl binding buffer (200 mM Tris–HCL, PH 8.0, 150 mM NaCl, 0.2% TritonX-100, 10% glycreol, 0.5 mM PMSF), and then the Resins were washed 5 times with wash buffer (500 mM Tris–HCL, PH 8.0, 140 mM NaCl, 0.1% TritonX-100, 1 mM EDTA) to remove non-specifically bound proteins, the precipitates were boiled in 1 × SDS loading buffer and detected by immunoblotting using corresponding antibodies.

### miRNA Northern blot analysis and qRT-PCR analysis

miRNA Northern blot was carried out as previously described [63]. Specifically, leaves of the rice seedlings were used for RNA extraction, and the OsmiR319 probe was synthesized with 3’-end Biotin. The blots were incubated at 42℃ for 30 min in the Hybridization Buffer (Ambion). And 50–80-pM probes were added in the hybridization buffer to incubate for one night. For RNA loading, 5S rRNA was used as control.

qRT-PCR analysis of miRNA was carried out as described [64], using miRNA 1st Strand cDNA Synthesis Kit (Vazyme Biotech, China) and miRNA Universal SYBR qPCR Master Mix (Vazyme Biotech, China). The 2^−ΔΔ*C*T^ method was employed to quantify relative gene expression levels. Mean of internal reference gene *U6* were used for normalization.

### Transcriptome analysis

For transcriptome analysis of *MYB*s after BPH infestation, seedlings of resistant variety RHT and moderately resistant variety ZH11 of about 45 days were infested by 15 s to third instar BPH nymphs. The leaf sheaths of each plant were collected 0, 12, and 24 h later, with three biological replicates per time-point. Total RNA was isolated using the TRIzol reagent (Invitrogen) according to the manufacturer’s instructions. The RNA was used to prepare libraries, which were sequenced on the BGISEQ-500 analyzer at BGI (Shenzhen).

### Primer sequences

All the oligo sequences used in this study are listed in Additional file 1W.

### Supplementary Information

**Additional file 1.** Raw data of the statistical charts in this study.

**Additional file 2.** Leaf phenotype of MIM319OE and miR319aOE plants.

**Additional file 3.** Small population assays of the miR319aOE, miR319bOE and two lines of MIM319OE plants as compared with the WT ZH11.

**Additional file 4.** BPH resistance tests of miR319 over expression in TP309 and Kasalath genetic backgrounds.

**Additional file 5.** Expression of putative miR319 target genes in miR319bOET (319bOE in the figure) and WT TP309.

**Additional file 6.** qRT–PCR detection of *OsPCF5* mRNA levels in PCF5OE and WT plants.

**Additional file 7.** Alignment of the TCP domain of the OsPCF5 protein with those of other bHLH proteins in rice (OsPCF5 is in the first line as indicated).

**Additional file 8.** Response of *MYB* genes to BPH infestation in different rice varieties.

**Additional file 9.** Pictures showing the result of small population assays of the MYB22OE-1 line and MYB22KO-1 plants as compared with ZH11 respectively.

**Additional file 10.** Original gels and blots.

## Data Availability

All data generated or analyzed during this study are included in this published article and its supplementary information files.

## Abbreviations

BPH: Brown planthopper
miRNAs: MicroRNAs
TCP: TEOSINTEBRANCHED1/CYCLOIDEA/PROLIFERATING CELL FACTOR
MYB: V-myb myeloblastosis viral oncogene homolog
JA: Jasmonate acid
GA: Gibberellin
*OsPAL*: *Phenylalanine ammonia-lyase*
*Os4CL*: 4-*coumarate:coenzyme A ligase*
SA: Salicylic acid
MIM: Target mimicry
Y2H: Yeast two-hybrid assay
LCA: Luciferase complementation assay
BIFC: Bimolecular fluorescence complementation
