# Supplementary material for: OsmiR319-OsPCF5 modulate resistance to brown planthopper in rice through association with MYB proteins
Source: BMC Biol. 2024 Mar 22;22:68. doi: 10.1186/s12915-024-01868-3 (PMC10960409; doi:10.1186/s12915-024-01868-3)
Supplement: Supplementary file 2 — Additional file 2. Leaf phenotype of MIM319OE and miR319aOE plants. [file 12915_2024_1868_MOESM2_ESM.docx]

**Additional file 2**


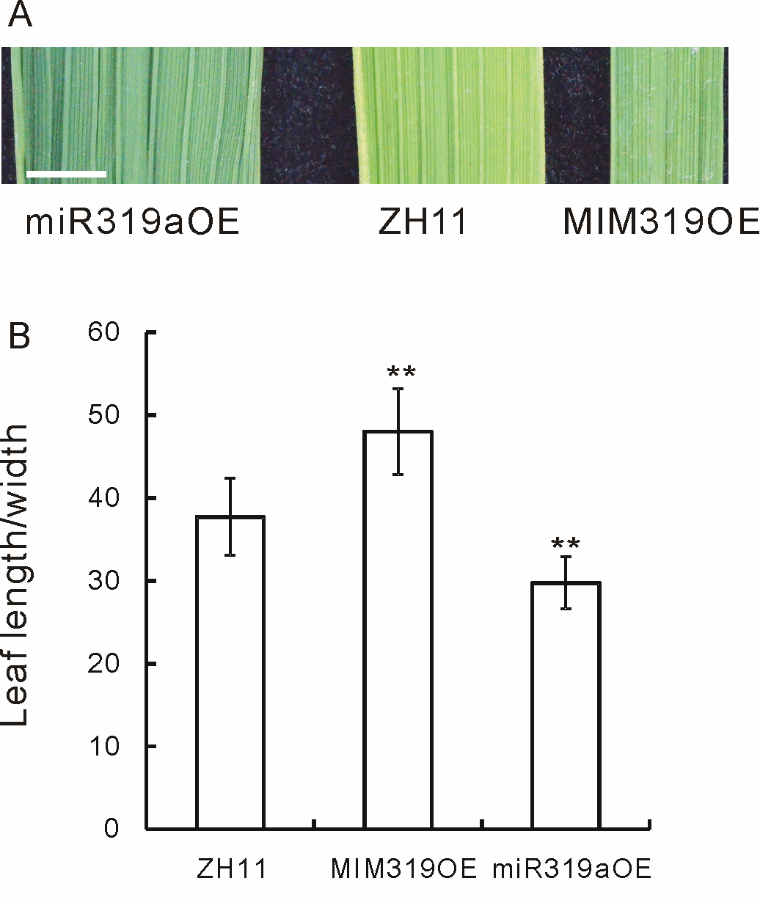


**Additional file 2 Leaf phenotype of MIM319OE and miR319aOE plants**

A, Leaf width of the MIM319OE plants as compared with that of the WT ZH11 and miR319aOE plants. Leaves in the picture are respective flag leaves, the bar was 5 mm. B, Ratio of the leaf length to width of the MIM319OE plant, ZH11 and miR319aOE plants. Data was collected from the first leaves from top at heading stage in summer of Shanghai. Double asterisks represented significant difference determined by the Student’s *t*-test at ***P*<0.01. Individual data values was provided in table S3.
