# Supplementary material for: OsmiR319-OsPCF5 modulate resistance to brown planthopper in rice through association with MYB proteins
Source: BMC Biol. 2024 Mar 22;22:68. doi: 10.1186/s12915-024-01868-3 (PMC10960409; doi:10.1186/s12915-024-01868-3)
Supplement: Supplementary file 3 — Additional file 3. Small population assays of the miR319aOE, miR319bOE and two lines of MIM319OE plants as compared with the WT ZH11. [file 12915_2024_1868_MOESM3_ESM.docx]

**Additional file 3**


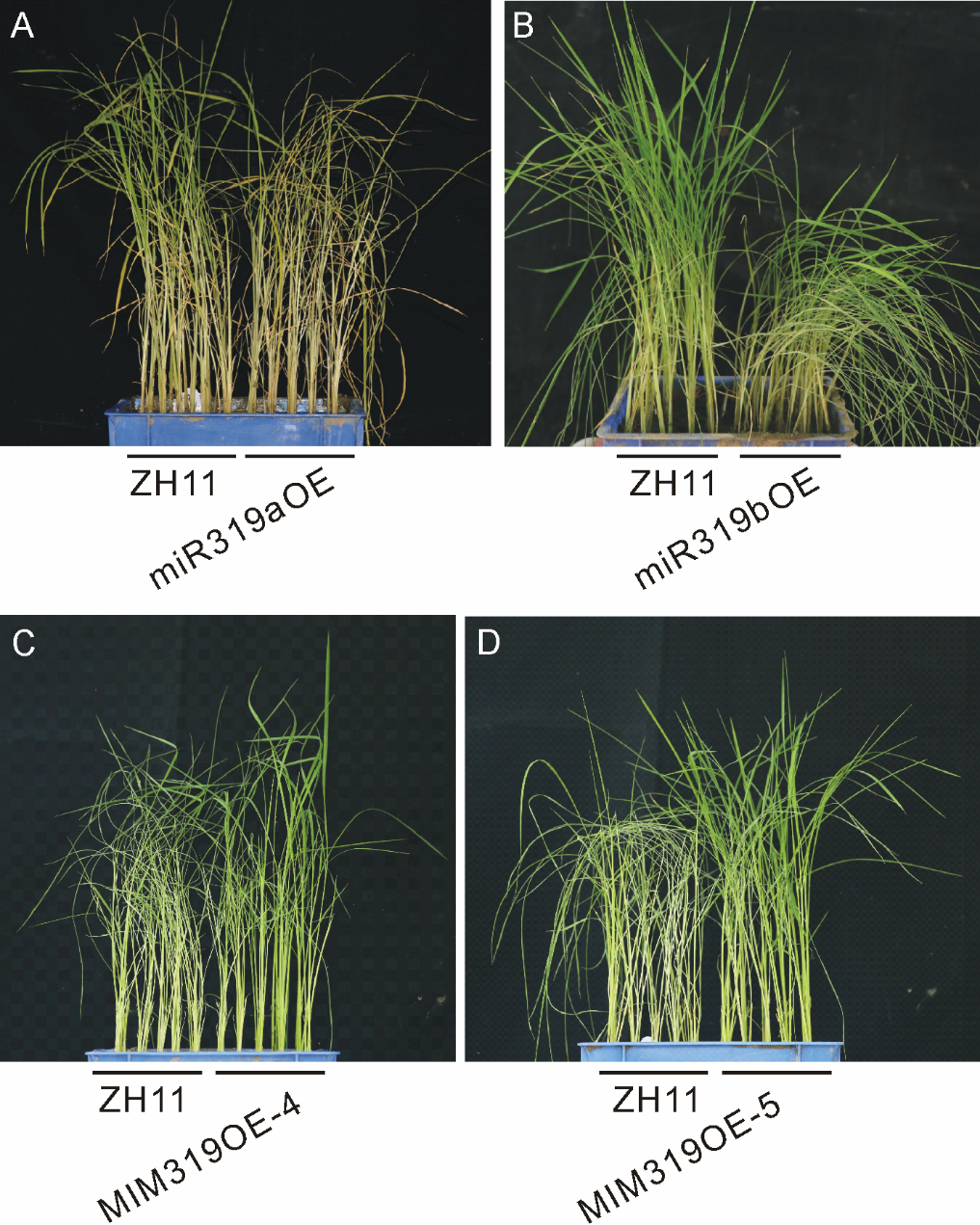


**Additional file 3 Small population assays of the miR319aOE, miR319bOE and two lines of MIM319OE plants as compared with the WT ZH11**

A, Small population assays of the miR319aOE and WT ZH11. B, Small population assays of the miR319bOE and WT ZH11. C, Small population assays of the MIM319OE-4 and WT ZH11. D, Small population assays of the MIM319OE-5 and WT ZH11. Pictures were taken after BPH infestation for about 7-10 day, when the two lines in each plate showed very obvious difference in status (death/alive) caused by BPH infestation.
