# Supplementary material for: OsmiR319-OsPCF5 modulate resistance to brown planthopper in rice through association with MYB proteins
Source: BMC Biol. 2024 Mar 22;22:68. doi: 10.1186/s12915-024-01868-3 (PMC10960409; doi:10.1186/s12915-024-01868-3)
Supplement: Supplementary file 4 — Additional file 4. BPH resistance tests of miR319 over expression in TP309 and Kasalath genetic backgrounds. [file 12915_2024_1868_MOESM4_ESM.docx]

**Additional file 4**

**
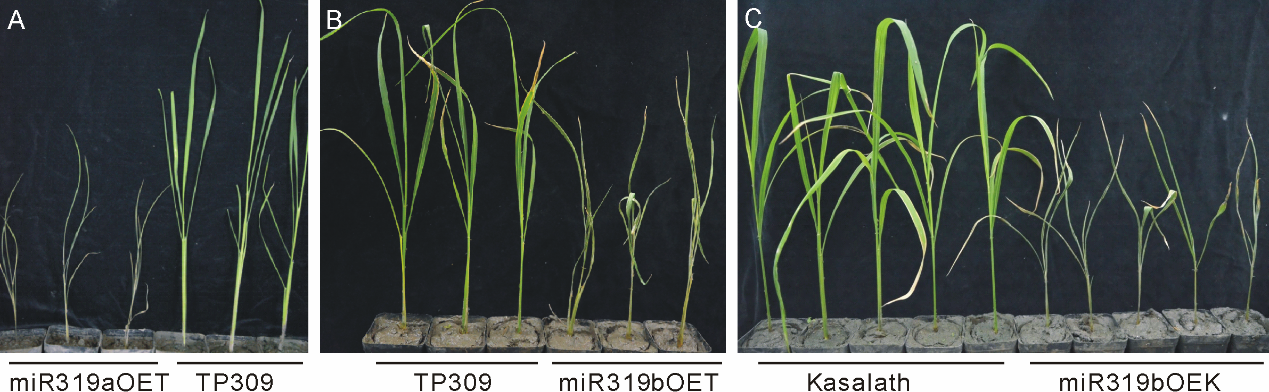
**

**Additional file 4 BPH resistance tests of miR319 over expression in TP309 and Kasalath genetic backgrounds as indicated**

A, Individual test of miR319a over expression in TP309 background (miR319aOET), in compassion with WT TP309. B, Individual test of miR319b over expression in TP309 background (miR319bOET), in compassion with WT TP309. C, Individual test of miR319b over expression in Kasalath background (miR319bOEK), in compassion with WT Kasalath.
