# Supplementary material for: OsmiR319-OsPCF5 modulate resistance to brown planthopper in rice through association with MYB proteins
Source: BMC Biol. 2024 Mar 22;22:68. doi: 10.1186/s12915-024-01868-3 (PMC10960409; doi:10.1186/s12915-024-01868-3)
Supplement: Supplementary file 5 — Additional file 5. Expression of putative miR319 target genes in miR319bOET (319bOE in the figure) and WT TP309. [file 12915_2024_1868_MOESM5_ESM.docx]

**Additional file 5**

**
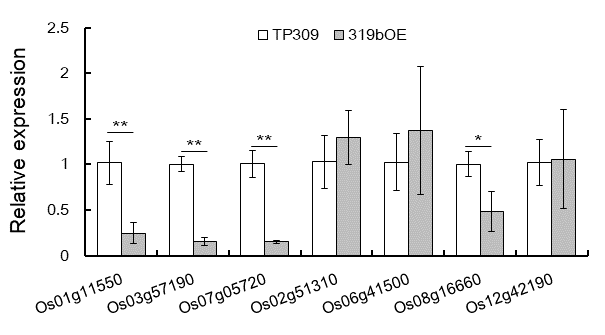
**

**Additional file 5 Expression of putative miR319 target genes in miR319bOET (319bOE in the figure) and WT TP309**

RNA samples of leaves for test were taken at seedling stage with three biological repeat. Asterisks represented significant difference as determined by the Student’s *t*-test at ***P*<0.01 and **P*<0.05. Individual data values was provided in table S7.
