# Supplementary material for: OsmiR319-OsPCF5 modulate resistance to brown planthopper in rice through association with MYB proteins
Source: BMC Biol. 2024 Mar 22;22:68. doi: 10.1186/s12915-024-01868-3 (PMC10960409; doi:10.1186/s12915-024-01868-3)
Supplement: Supplementary file 6 — Additional file 6. qRT–PCR detection of OsPCF5 mRNA levels in PCF5OE and WT plants. [file 12915_2024_1868_MOESM6_ESM.docx]

**Additional file 6**

**
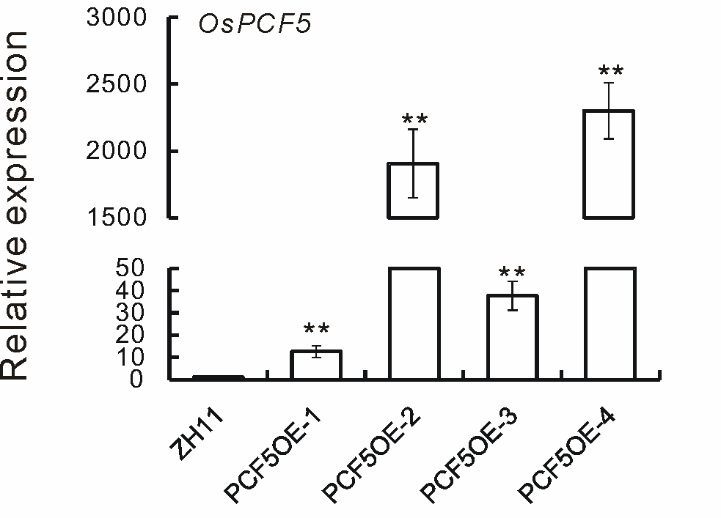
**

**Additional file 6 qRT**–**PCR detection of *OsPCF5* mRNA levels in PCF5OE and WT plants**

RNA samples of leaves for test were taken at seedling stage with three biological repeat. Asterisks represented significant difference compared with WT ZH11 as determined by the Student’s *t*-test (**, *P*<0.01; **P*<0.05). Individual data values was provided in table S10.
