# Supplementary material for: OsmiR319-OsPCF5 modulate resistance to brown planthopper in rice through association with MYB proteins
Source: BMC Biol. 2024 Mar 22;22:68. doi: 10.1186/s12915-024-01868-3 (PMC10960409; doi:10.1186/s12915-024-01868-3)
Supplement: Supplementary file 7 — Additional file 7. Alignment of the TCP domain of the OsPCF5 protein with those of other bHLH proteins in rice (OsPCF5 is in the first line as indicated). [file 12915_2024_1868_MOESM7_ESM.docx]

**Additional file 7**

**
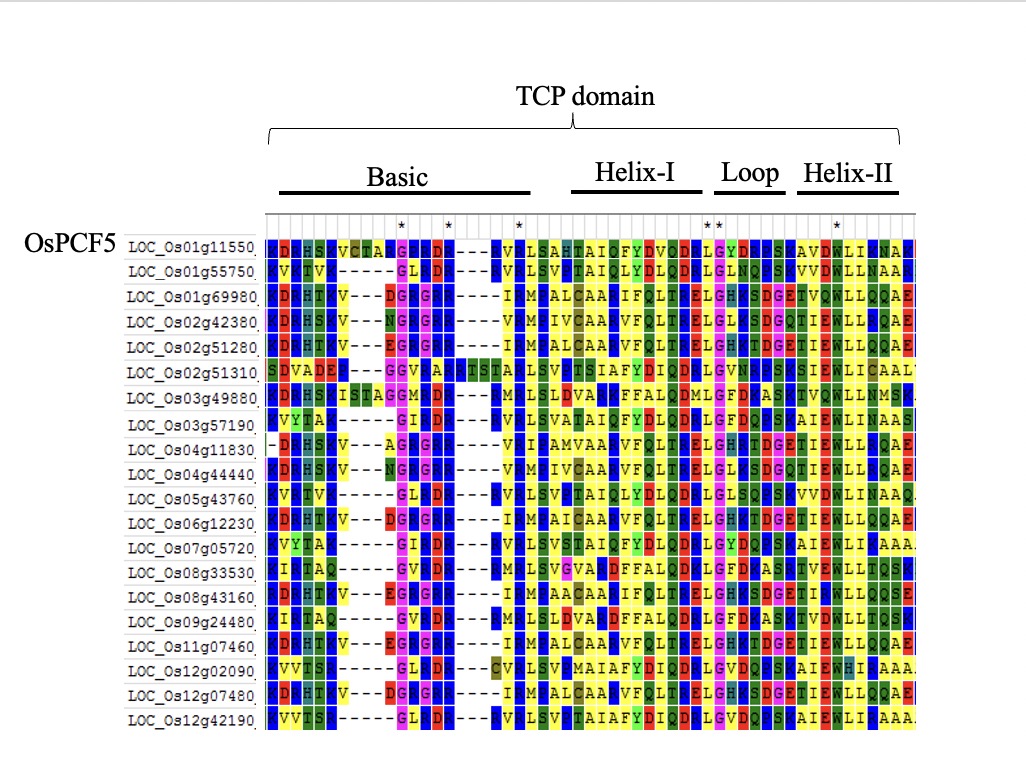
**

**Additional file 7 Alignment of the TCP domain of the OsPCF5 protein with those of other bHLH proteins in rice (OsPCF5 is in the first line as indicated)**

Regions in display are only the TCP domain of respective proteins. Asterisks represented the amino acids which are the same in all the proteins analyzed.
