# Supplementary material for: OsmiR319-OsPCF5 modulate resistance to brown planthopper in rice through association with MYB proteins
Source: BMC Biol. 2024 Mar 22;22:68. doi: 10.1186/s12915-024-01868-3 (PMC10960409; doi:10.1186/s12915-024-01868-3)
Supplement: Supplementary file 8 — Additional file 8. Response of MYB genes to BPH infestation in different rice varieties. [file 12915_2024_1868_MOESM8_ESM.docx]

**Additional file 8**

**
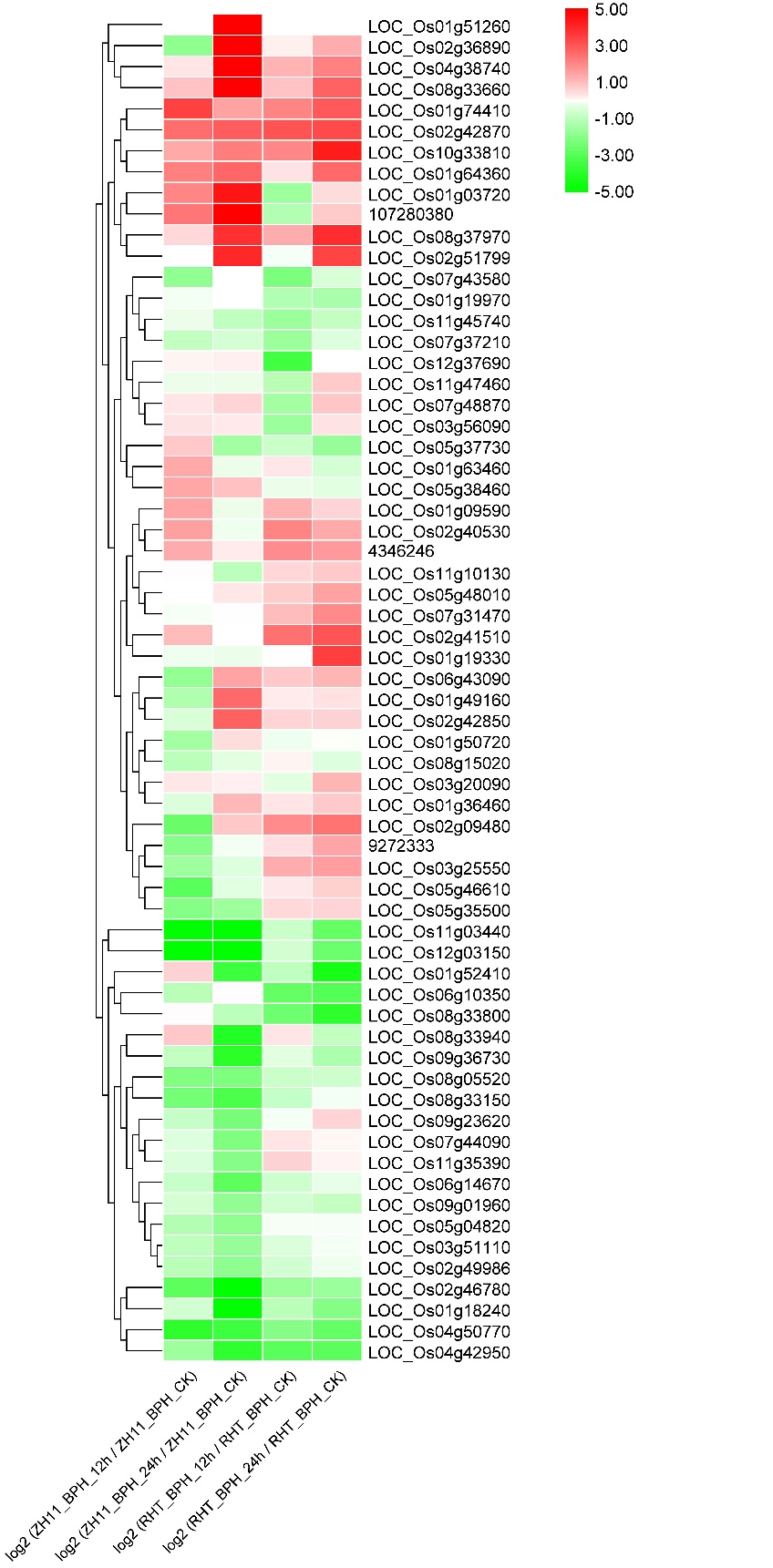
**

**Additional file 8 Response of *MYB* genes to BPH infestation in different rice varieties.**

The resistant variety RHT and the moderately resistant variety ZH11 were infested by BPH at 0h, 12h and 24h. The log2 (fold-change) data from three biological replicates of 12h vs 0h, and 24h vs 0h was respectively used to draw heat map using TBtools software. The Gene ID from NCBI or MSU presented different *MYB* genes. Individual data values was provided in table S13.
