# Supplementary material for: OsmiR319-OsPCF5 modulate resistance to brown planthopper in rice through association with MYB proteins
Source: BMC Biol. 2024 Mar 22;22:68. doi: 10.1186/s12915-024-01868-3 (PMC10960409; doi:10.1186/s12915-024-01868-3)
Supplement: Supplementary file 9 — Additional file 9. Pictures showing the result of small population assays of the MYB22OE-1 line and MYB22KO-1 plants as compared with ZH11 respectively. [file 12915_2024_1868_MOESM9_ESM.docx]

**Additional file 9**

**
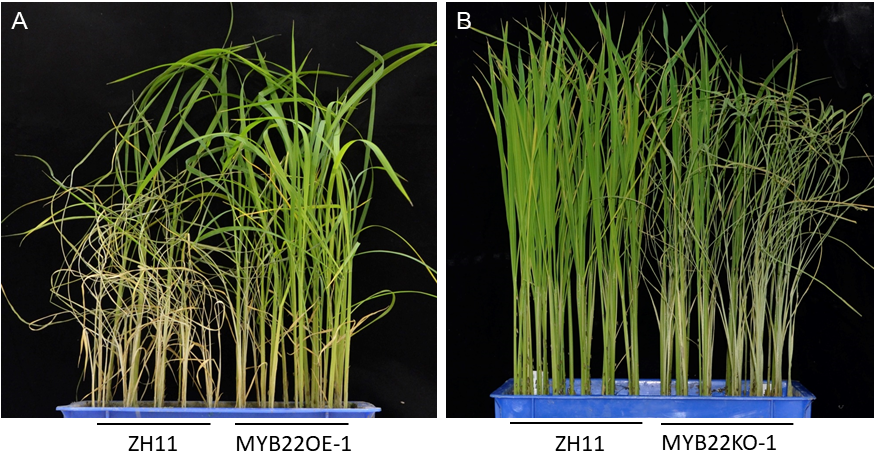
**

**Additional file 9 Pictures showing the result of small population assays of the MYB22OE-1 line and MYB22KO-1 plants as compared with ZH11 respectively**

A, Small population assay of MYB22OE-1 and ZH11. B, Small population assay of MYB22OE-1 and ZH11. Pictures were taken at 7-12 days after BPH infestation when obvious difference of the status (death/alive) of the two lines in the same plate could be observed.
