# Supplementary material for: OsmiR319-OsPCF5 modulate resistance to brown planthopper in rice through association with MYB proteins
Source: BMC Biol. 2024 Mar 22;22:68. doi: 10.1186/s12915-024-01868-3 (PMC10960409; doi:10.1186/s12915-024-01868-3)
Supplement: Supplementary file 10 — Additional file 10. Original gels and blots. [file 12915_2024_1868_MOESM10_ESM.docx]

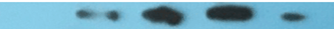


Original Figure 1B up


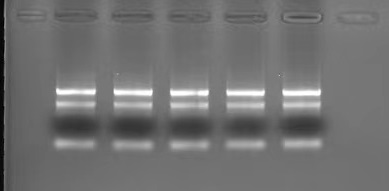


Original Figure 1B down


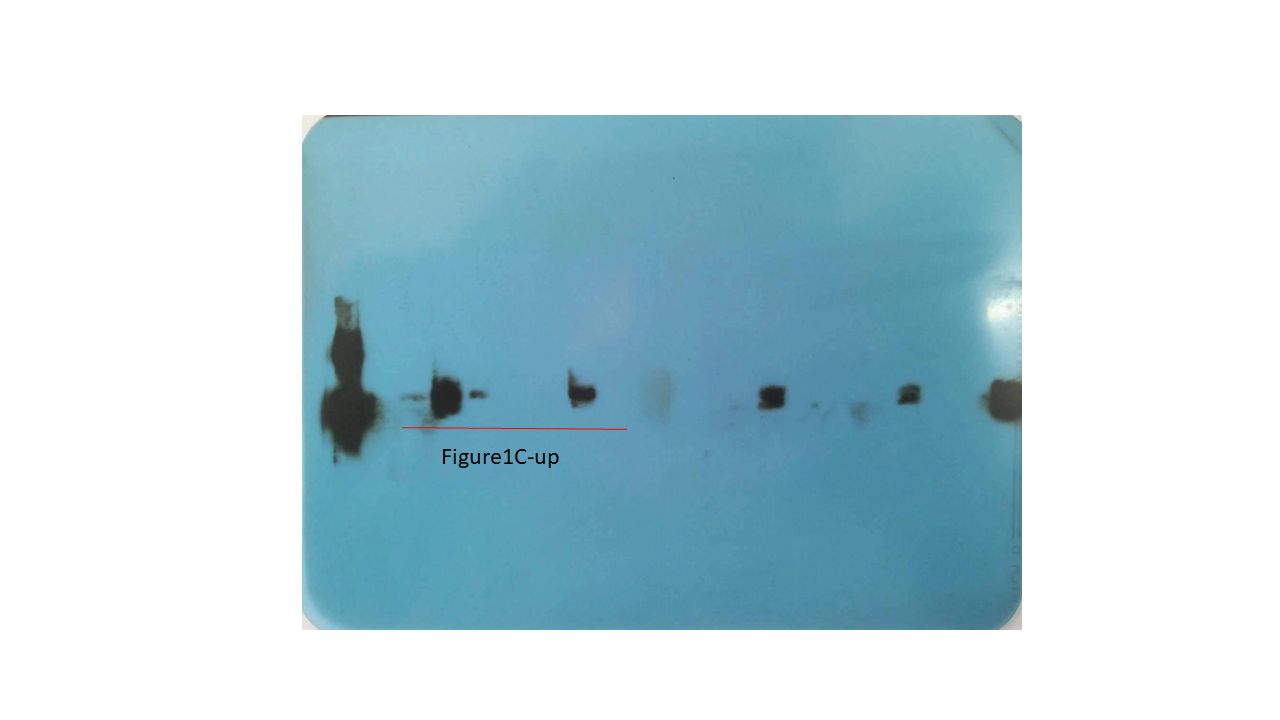


Original Figure 1C up


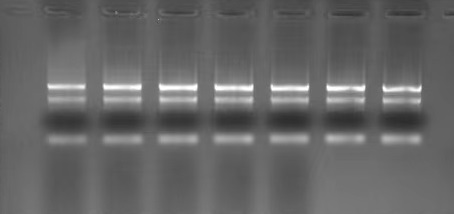


Original Figure 1C down


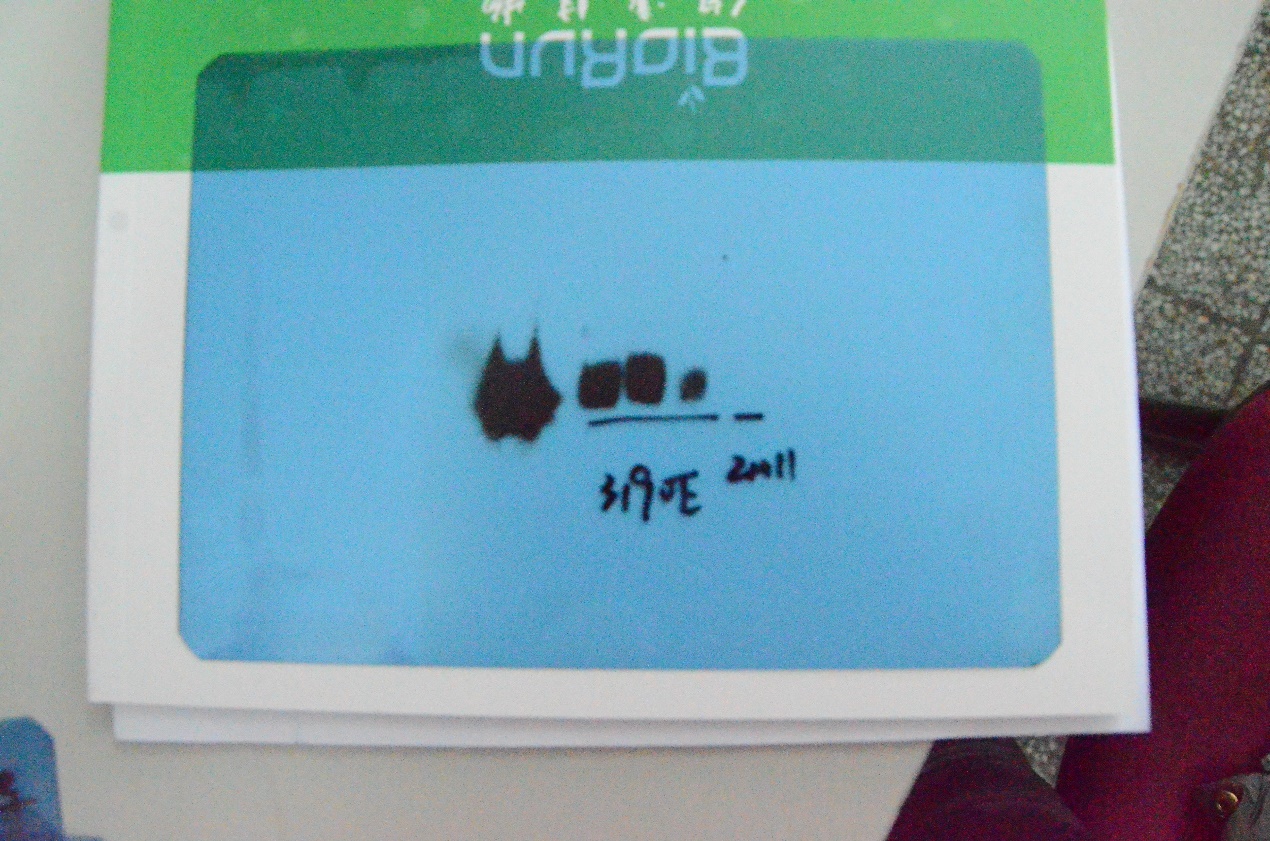


Original Figure 1D up


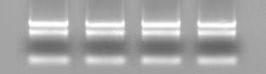


Original Figure 1D down


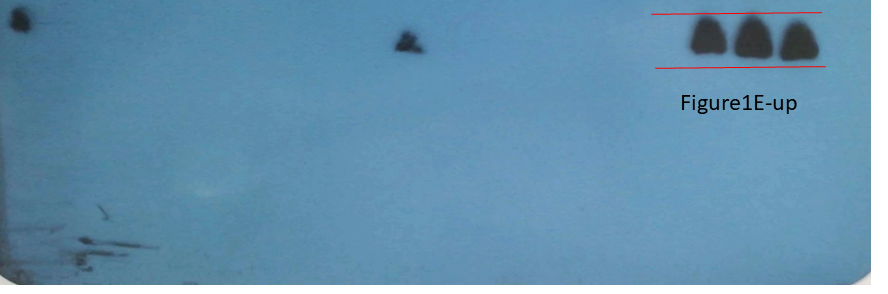


Original Figure 1E up


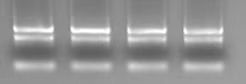


Original Figure 1E down


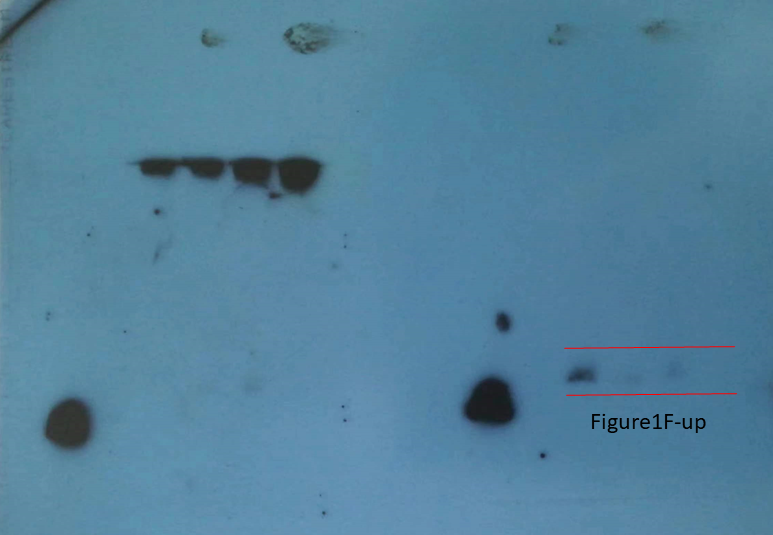


Original Figure 1F up


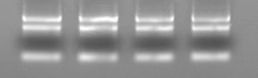


Original Figure 1F down


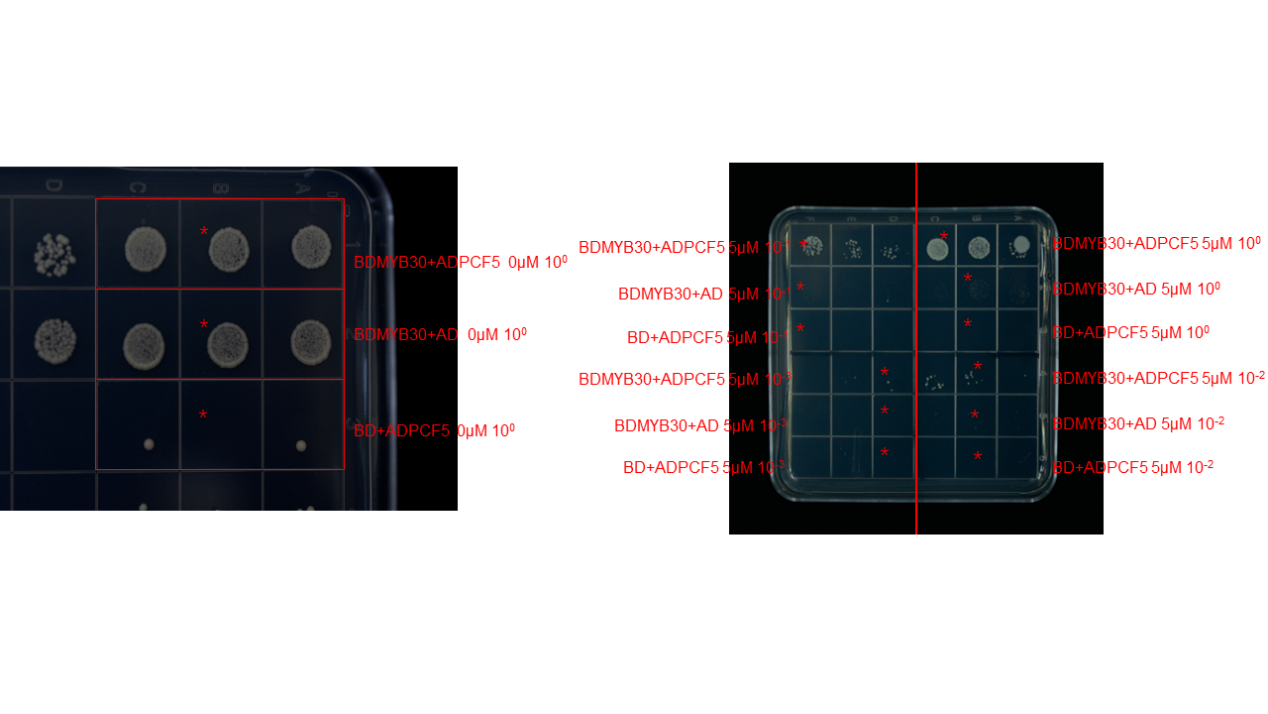


Original Figure 3A


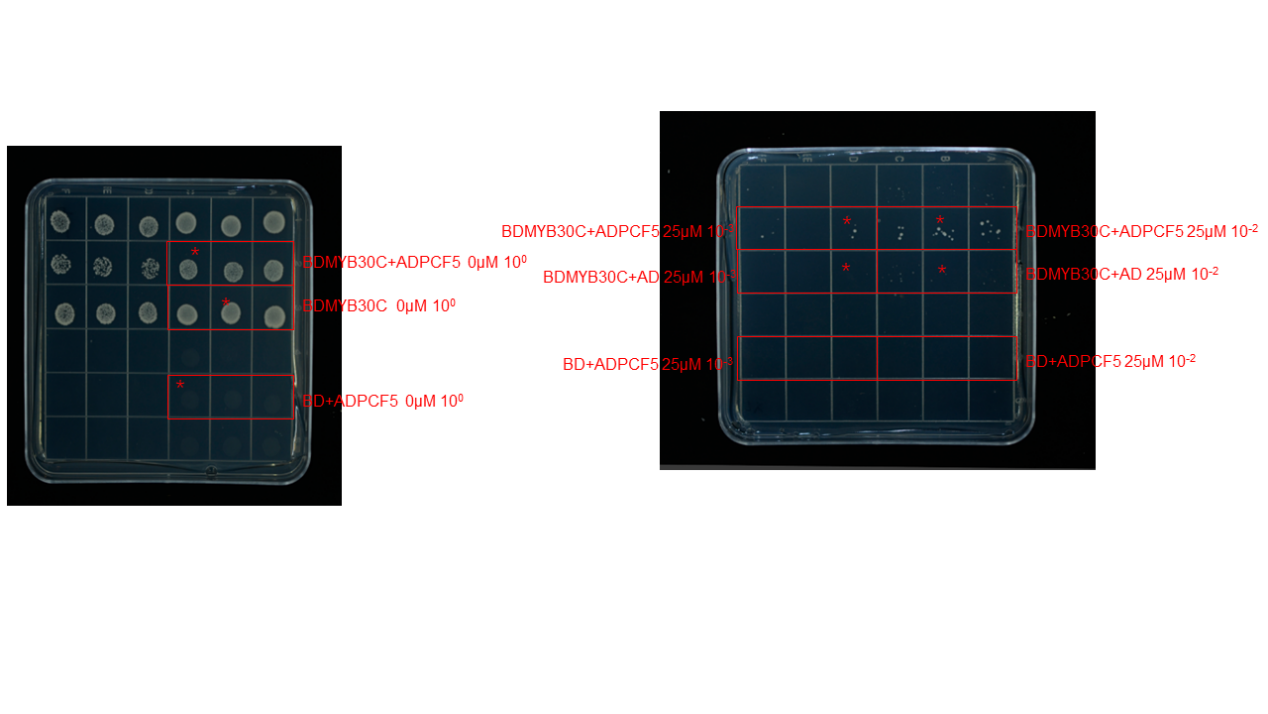
 Original Figure 3D


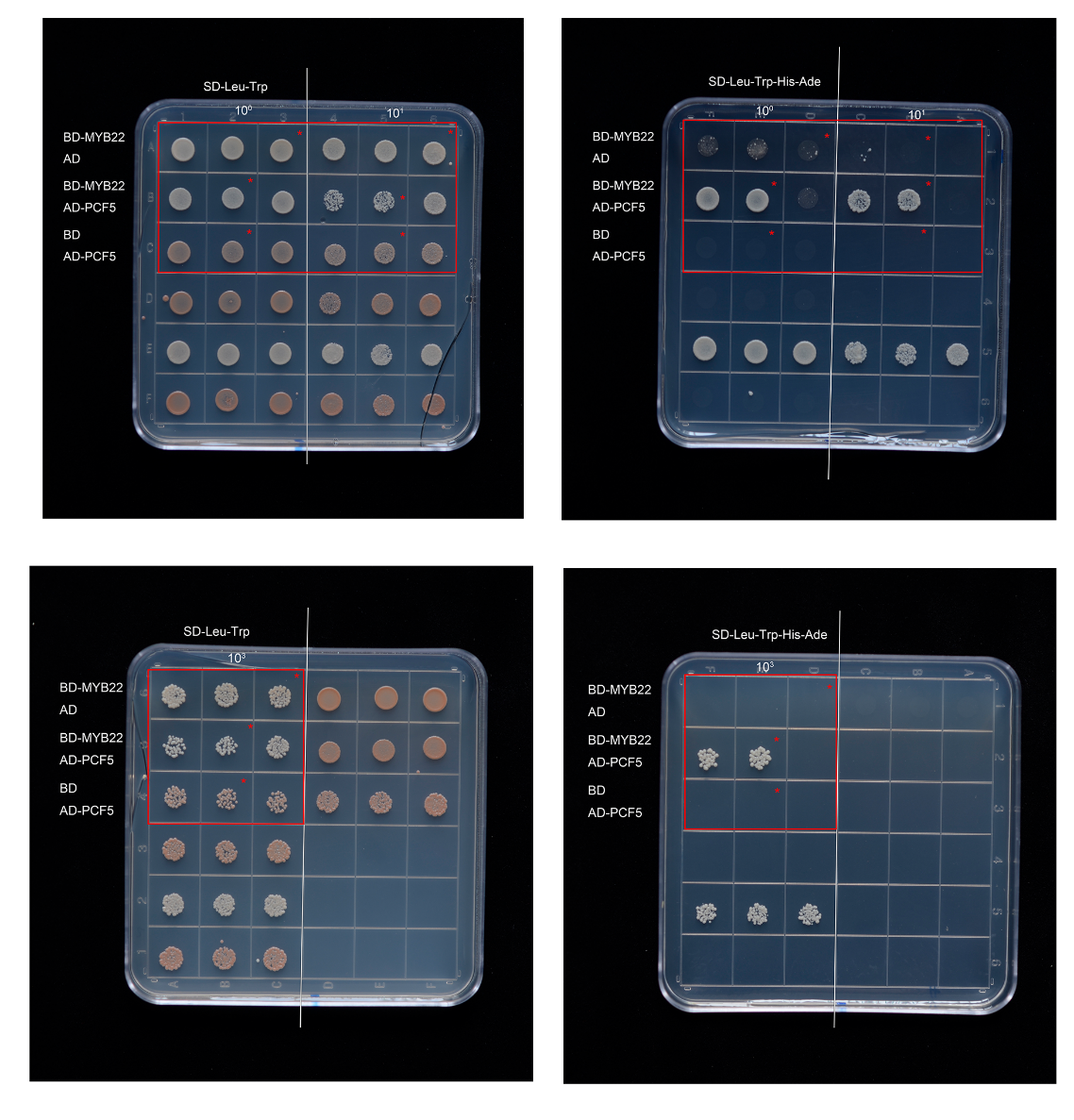


Original Figure 4A


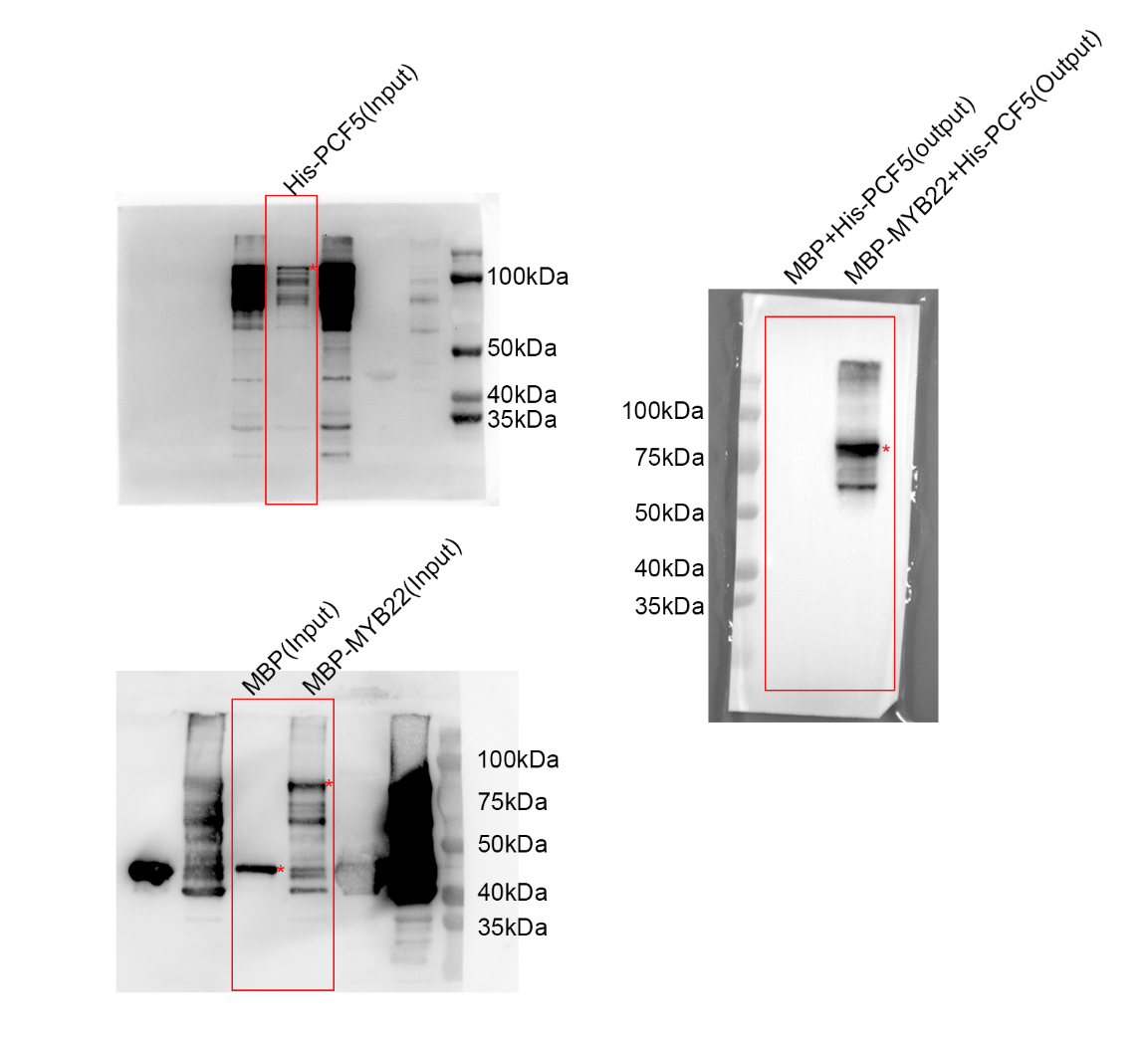


Original Figure 4D
